# How cyanophage S-2L rejects adenine and incorporates 2-aminoadenine to saturate hydrogen bonding in its DNA

**DOI:** 10.1038/s41467-021-22626-x

**Published:** 2021-04-23

**Authors:** Dariusz Czernecki, Pierre Legrand, Mustafa Tekpinar, Sandrine Rosario, Pierre-Alexandre Kaminski, Marc Delarue

**Affiliations:** 1 Unit of Structural Dynamics of Biological Macromolecules, CNRS UMR 3528, 25-28 rue du Docteur Roux, Institut Pasteur, Paris, France; 2 Sorbonne Université, Collège Doctoral, ED 515, Paris, France; 3 Synchrotron SOLEIL, L’Orme des Merisiers, Saint Aubin, Gif-sur-Yvette France; 4 Unit of Biology of Pathogenic Gram-Positive Bacteria, 25-28 rue du Docteur Roux, Institut Pasteur, Paris, France

**Keywords:** X-ray crystallography, DNA metabolism, X-ray crystallography

## Abstract

Bacteriophages have long been known to use modified bases in their DNA to prevent cleavage by the host’s restriction endonucleases. Among them, cyanophage S-2L is unique because its genome has all its adenines (A) systematically replaced by 2-aminoadenines (Z). Here, we identify a member of the PrimPol family as the sole possible polymerase of S-2L and we find it can incorporate both A and Z in front of a T. Its crystal structure at 1.5 Å resolution confirms that there is no structural element in the active site that could lead to the rejection of A in front of T. To resolve this contradiction, we show that a nearby gene is a triphosphohydolase specific of dATP (DatZ), that leaves intact all other dNTPs, including dZTP. This explains the absence of A in S-2L genome. Crystal structures of DatZ with various ligands, including one at sub-angstrom resolution, allow to describe its mechanism as a typical two-metal-ion mechanism and to set the stage for its engineering.

## Abstract (web summary)

The cyanophage S-2L incorporates 2-aminoadenine (Z) instead of adenine (A) in its genome. Here, the authors provide an explanation for the absence of A in S-2L genome by identifying and characterising functionally and structurally both the HD phosphohydrolase (*datZ*) that specifically cleaves dATP, and the sole DNA primase-polymerase of S-2L, nonspecific of dATP or dZTP.

## Introduction

All living organisms use the same elementary bricks for their genetic material, namely four, and only four, nucleobases: adenine (A), thymine (T), guanine (G) and cytosine (C). However, certain viruses of bacteria (bacteriophages or phages) use modified bases to escape their host’s defence system, especially their endonucleases^1,2^. Most of the observed DNA modifications occur at position 5 of pyrimidines or position 7 of purines that face the major groove of the DNA double helix^1,3^. Methylation on N4 of cytosine or N6 of adenine are also observed in viruses^2,4^. For pyrimidines, DNA containing 5-hydroxymethylcytosine has long been known to exist in phages T2, T4 and T6^5^, along with the enzyme (deoxycytidylate hydroxymethylase) responsible for its biosynthesis^6^; more complicated post-replicative pathways of thymine hypermodification were recently found in phages and recreated in vitro^7^. For purines, archaeosine, a modified 7-deaza analogue of guanine observed in archaeal tRNA D-loop^8^ was found in the genome of the *E. coli* siphophage 9 g^9^, and is possibly present in another siphophage BRET^10^; their genomes encode genes (QueC, QueD, QueE) necessary for the biosynthesis of guanine modification. Recently, three additional 7-deazaguanine analogues have been identified and characterised in the genomes of phages and archaeal viruses^11^. An important point is to distinguish between replicative and post-replicative DNA modifications: if a biosynthetic pathway can be identified for the synthesis of the triphosphate of the modified nucleotide, it is reasonable to assume that the modified base is incorporated during replication and is not the result of a post-replicative modification.

**Fig. 1:**
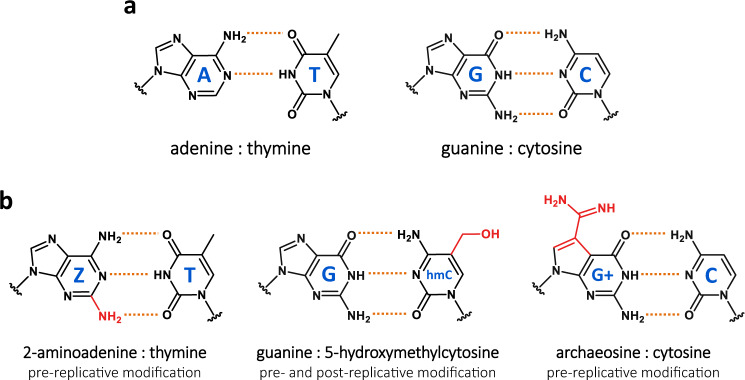
Watson–Crick base pairs and natural variations thereof. Hydrogen bonds are marked by a dotted orange line. **a** Classical DNA base pairs, universal to all three domains of life and most viruses. **b** Other types of base pairs with three hydrogen bonds found in some organisms and viruses. Additional chemical groups are in red. 2-aminoadenine : thymine (Z:T, left); guanine : 5-hydroxymethylcytosine (G:hmC, centre); archaeosine : cytosine (G + :C, right). The Z:T pair, first found in cyanophage S-2L, replaces completely the usual A:T pair in the genome.

Cyanophage S-2L is a Synechococcus phage from the double-stranded DNA *Siphoviridae* family. It was first isolated and described in 1977^12^ and its genome was shown to contain no adenine nor any of its 7-deaza derivatives. Instead, it uses 2-aminoadenine (2,6-diaminopurine or Z) that has an additional amino group in position 2 compared to adenine^13^. The A:T base pair, with two hydrogen bonds, is therefore replaced by the Z:T base pair that has three hydrogen bonds, as in the G:C base pair (Fig. 1). This feature, combined with an unusually high GC content of S-2L genome, explains its exceptionally high melting point^12^. It is believed that the A-to-Z substitution arose as a form of host evasion tactics, rendering S-2L’s DNA resistant to the DNA-targeting proteins of its host, especially endonucleases^14,15^.

Once the S-2L’s genome was sequenced, the presence of a gene homologous to an adenylosuccinate synthetase (*purA*) was noted, raising the possibility that the phage encodes in its genome the enzymes of the biosynthesis pathway of 2-aminoadenine triphosphate (dZTP; patent application EP1499713A2). A detailed structural study of such *purA* orthologue (called *purZ*) in vibriophage φVC8 fully supported this hypothesis (PDB ID: 6FM1). However, it remained still largely unknown how the phage S-2L incorporates the base Z in its genome, especially as no gene corresponding to a DNA polymerase could be detected. This is in contrast with the situation in the phage φVC8, where a DNA polymerase of the family A has been identified^16^.

Here, we identify the enzyme that is responsible for genome duplication of the phage S-2L, a member of the PrimPol family, and we present its crystal structure. We confirm its polymerase activity but find that the enzyme is not specific to A or Z. Instead, we propose that the absence of A in S-2L genome is explained by a separate enzyme, an HD phosphohydrolase that specifically dephosphorylates dATP and that we name DatZ. We give a structural explanation for both the specificity and the reaction mechanism of DatZ, based on three crystallographic structures, including one determined at sub-angstrom resolution.

## Results

### A DNA primase-polymerase nonspecific of A or Z

Parsing the genomic sequence of cyanophage S-2L (AX955019) in the search for a protein involved in DNA replication, we identified one ORF corresponding to a member of the Archaeo-Eukaryotic Primase (AEP) superfamily, which had not been noted earlier. We will refer to it as “PrimPol”, similarly to its close homologues, and its gene will be referred to as “*pplA*”.

AEP is the eukaryotic and archaeal counterpart of DnaG, the bacterial primase superfamily^17,18^, to which it is structurally unrelated. Its members are found in all domains of life, including viruses, and are involved in several DNA transactions including not only DNA priming and replication, but also DNA repair through non-homologous end-joining (NHEJ)^18^. AEP proteins are often fused or physically interact with DNA helicases, and also with partners containing helix bundle domains (like PriCT-1, PriCT-2, PriL or PriX) that interact with the template ssDNA^17,19–22^. Particularly important for this work, it was recently shown that a phage-encoded AEP polymerase is capable of replicating the whole genome of the NrS-1 phage^23^. Although AEP is not officially included yet in the standard DNA polymerase classification encompassing polymerases from families A, B, C, D, X, Y and RT^24,25^ despite an incentive to do so^18^, members of the AEP superfamily share the classical Klenow fold with families A, B and Y DNA polymerases^26^.

**Fig. 2:**
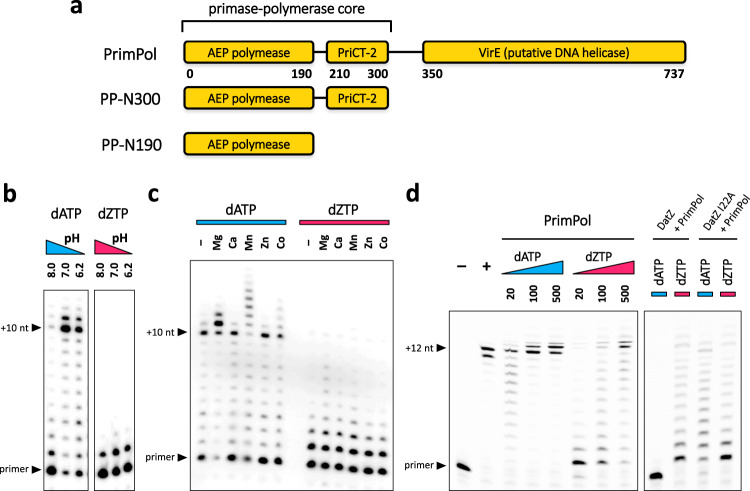
Functional characterisation of S-2L PrimPol. **a** Schematic diagram of S-2L PrimPol constructs showing its different domains with their respective amino-acid range (to scale). **b**–**d** Results of DNA polymerase activity tests of S-2L PrimPol with either dATP (blue) or dZTP (magenta) as the incoming dNTP, using templates with dT_10_GG (**b** and **c**) or dT_12_ (**d**) overhang. **b** Different buffers with various pHs, noted below the coloured triangles. **c** Effect of different divalent ions, at 5 μM each. **d** Effect of growing concentrations of nucleotides (lanes 3–8) and pre-incubation of reactional mixture for DatZ WT (lanes 9−10) and I22A mutant (lanes 11−12). Nucleotide concentrations are given in μM under the coloured triangles on the panel to the left; unless otherwise stated they are at 500 μM. Lanes 1–2 represent, respectively, a negative control without any polymerase, and a positive control with *E. coli* Pol I (Klenow fragment) and dATP.

We started by characterising the domain organisation of PrimPol in silico, using DISOPRED^27^. The result indicated that the enzyme is composed of three domains, whose function was then determined individually by homology searches (Fig. 2a). The first region (1–190) corresponds to the AEP domain itself, with all crucial motifs conserved. The second region (210–300) has a strong homology with PriCT-2 domain, most probably involved in the priming activity^19^. Together they are joined by a flexible linker and form the primase-polymerase component (1–300). The C-terminal domain (350–737) begins after another large flexible linker. BLAST searches^28^ indicated it matches best the VirE family of single-stranded DNA-binding proteins of function not described in the literature^29^. However, homology detection combined with structure prediction performed with HHpred^30^ found high-scoring similarity between viral hexameric DNA helicase structures, the closest being from bovine papillomavirus (2GXA).

We found no other detectable DNA polymerase in the S-2L’s genome and went on to assay the DNA polymerase activity of PrimPol. Specifically, we looked for its ability to selectively incorporate the base Z in front of an instructing base T, discarding the dATP present in the host cell’s dNTP pool and avoiding the A:T base pair altogether. We cloned and overexpressed the synthetic gene of PrimPol in *E. coli* and tested the product’s polymerase activity in vitro. To study the specificity towards A and Z, we used dsDNA with a dT_12_ oligomer as the 5′ overhang of the template strand and either dATP or dZTP in the reactional mixture. We tested a range of different conditions, varying temperature, pH, DNA, nucleotide and enzyme concentrations, as well as divalent ions (Fig. 2b–d) that are usual cofactors in DNA and RNA polymerases^31^. All assays indicate that S-2L PrimPol is capable of incorporating both nucleotides across from T, accepting A more readily than Z. We also noted that the presence of Mn^2+^ ions induces limited terminal transferase activity, as observed for some other DNA polymerases such as the human pol μ from the pol X family^32^; for another, more distantly related AEP, this activity was observed even with Mg^2+^ ions^33^. We also overexpressed truncated versions of the enzyme, PP-N300 and PP-N190, corresponding to the primase-polymerase core and polymerase domain, respectively. We observed a gradual decrease in the polymerase activity with progressive domain deletions, but constructs remain active as long as the AEP domain is present (Supplementary Fig. 1a); this confirms the necessary and sufficient role of this domain during DNA synthesis. In another test, we showed that PP-N300 can synthesise in vitro the first 124 nucleotides of its own native gene, with both dATGC and dZTGC mixtures (Supplementary Fig. 1b).

### Structural analysis of the AEP domain of S-2L PrimPol

**Fig. 3:**
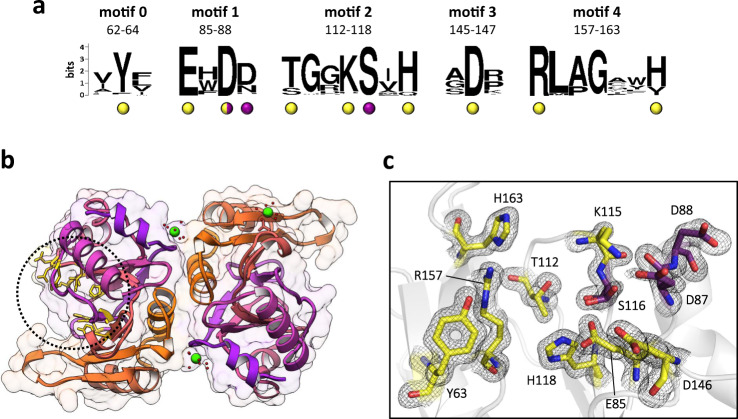
AEP domain of S-2L PrimPol: conserved residues and their structural context. **a** Five AEP motifs of PP-N190 close homologues. In addition to previous motif classifications^19,37^, the steric gate tyrosine is included as motif 0, and motifs 1 and 2 are extended. Numbers on top of the sequence blocks indicate their amino acid range according to S-2L PrimPol. Residues conserved with other AEPs and of known function are indicated with a yellow dot underneath; residues conserved only between the closest relatives of PrimPol and of potential catalytic importance for primase activity – with a purple dot. The double-hatted residue D87 could be involved in both polymerase (known) and/or primase (suggested) activities. **b** Structure of PP-N190 in ribbon and surface representation, with two symmetric molecules in the crystallographic asymmetric unit, each coloured with an orange–purple gradient. Calcium ions are shown by green spheres, with water molecules forming their hydration shells shown as red ones. The catalytic site of molecule A is shown in yellow stick representation and indicated with a dotted circle. **c** Zoom on the catalytic site of PP-N190. Residues highlighted in **a** are shown in stick representation and labelled, maintaining the same colour code. The experimental 2F_o_–F_c_ electron density around these residues (black mesh) is contoured at 1 sigma.

Using BLAST, we identified 129 other sequences with high similarity to the AEP domain of PrimPol (PP-N190). We aligned them and visualised the conservation status of crucial residues and motifs described in previous reports (Fig. 3a); their function is described further below.

We could crystallise PP-N190 and solve its structure at 1.5 Å resolution (PDB ID: 6ZP9; Supplementary Table 1 and Fig. 3b), using phase information from SeMet derivative crystals. Ca^2+^ ions were mandatory in the mother liquor to obtain crystals. As expected, the protein has a classical AEP fold. All crucial residues cluster together in the catalytic site of the domain (Fig. 3c). Y63, E85, D87, T112, K115, H118, D146, R157 are conserved across all AEPs (or have biochemically similar counterparts), and their function is well established in the superfamily. Residue Y63 plays the role of a steric gate for ribonucleotides, allowing only dNTPs in the catalytic site^34^. Residues E85, D87 (that can vary to Asp and Glu, respectively) coordinate a divalent metal ion (M^2+^) in the B site, that positions the triphosphate of the incoming nucleotide (dNTP) during polymerisation; this triphosphate is further stabilised by interactions with T112, K115, H118 and R157 (possibly varying respectively to Ser, Arg, Asn and Lys)^35–38^. Residue D146 along with residues E85, D87 and the dNTP’s α-phosphate coordinate another M^2+^ ion in the A site, making it possible to add the incoming dNTP to the primer strand of the nascent nucleic acid through the two-metal-ion mechanism^35,39,40^. The three negatively charged residues E85, D87 and D146 are crucial for the polymerase and primase activity, as shown in the related human PrimPol^41^. Importantly, in S-2L PP-N190 we noticed a significant positional shift of residue D87 compared to other AEP structures, along with the conservation among the close relatives of the neighbouring residue D88, which is exposed to the solvent. Either D87 is able to come back to its canonical position once all the substrates and ions are in place, or its position is conserved in the complex: to resolve this point, we investigate below with molecular dynamics its flexibility and potential to stabilise an additional metal ion together with D88. Finally, although residue H163 lies further apart from the triphosphate, its high conservation and covariance with positions R157 and H118 was noticed in a recent study^19^. In human PriS, the mutation of the corresponding residue (H324) to alanine partially inhibited the enzymatic activity, a result that was explained by the presence of a water molecule that links it to the triphosphate^36^.

Due to the presence of divalent calcium ions in all crystallisation conditions, we could not soak the crystals with nucleotides which immediately precipitate; transferring crystals to a solution devoid of Ca^2+^ dissolved them in a matter of seconds. On the other hand, there are several AEP structures with bound ligands available in the PDB, including DNA and (d)NTPs. Based on the three structures with DNA (3H25, 3PKY, 5L2X), the nucleic acid apparently bends in an L-shape over the open catalytic site (Supplementary Fig. 2a). Additionally, the incoming (d)NTP’s conformation is largely conserved across all eight unique AEP structures with a bound nucleotide (PDB IDs: 1V34, 2ATZ, 2FAQ, 3PKY, 5L2X, 5OF3, 6JON, 6R5D). In all cases, the catalytic site is open to the solvent and there is no selection on the incoming nucleotides; after superposition with these structures, PP-N190 presents no structural feature that could lead to a Z vs A specificity during the polymerase reaction.

### In silico investigation of the primase catalytic site

In standard primase assays involving a typical single-stranded M13 genome or several random oligonucleotide sequences (50–100 nt), we observed no DNA or RNA primase activity of PrimPol, perhaps because of incompatible template sequence. Nevertheless, using computer simulations, we tried to understand how PrimPol may work in the primase mode, a function that is predicted to be conserved in the enzyme by high homology to other active primase-polymerases. Relying on structure of human PrimPol^37^, we could build a model of S-2L PrimPol AEP domain with a Mg^2+^ ion placed in the classical site B in the presence of two nucleotide triphosphates in the elongation (polymerase) and initiation (primase) sites. We placed an additional Mg^2+^ ion in a hypothetical metal binding site “C” between residues D87 and D88 (Supplementary Fig. 2b). Using this initial model, we conducted molecular dynamics simulations to investigate the stability of the complex in the catalytic site.

We observed during these simulations that the side chain of S116 was coordinating the Mg^2+^ ion in the B site, together with the usually involved residue E85 (Supplementary Fig. 2c). Strictly conserved between closely related PP-N190 relatives but not across the AEP superfamily, S116 can apparently take over the function of the shifted D87 residue, rather than contacting the γ-phosphate of the incoming nucleotide as seen for its counterpart in human PrimPol^37^. Additionally, the Mg^2+^ ion placed at site C between residues D87 and D88 was stable during the 212 ns-long MD simulation, and interacts with the γ-phosphate of the nucleotide in the initiation site. The possible change of D88 to Asn or to His observed in related AEP domains retains the capacity of divalent metal ion binding and further supports the functional nature of this position. We propose that during the putative primase activity of PrimPol involving two nucleotide triphosphates, this additional ion binding site C is important in the positioning and charge neutralisation of the 5′ nucleotide. To test this hypothesis, further work is needed to find the sequence of the template that triggers the DNA primase activity. Then, site-directed mutagenesis can be used to probe the role of putative important residues pointed out by our model.

In conclusion, while the discovery of PrimPol encoded in S-2L’s genome explains how the phage could replicate its genome, functional and structural studies show it cannot discriminate A against Z. Therefore, it remains to be explained how Z gets incorporated in the genome of S-2L instead of A.

### DatZ: a triphosphohydrolase specific of dATP

We subsequently revisited other genes susceptible to intervene during the phage genome replication. We found that one ORF in the immediate vicinity of *purZ* encodes a 175 aa protein belonging to the HD-domain phosphohydrolase family^42^. Enzymes from this family are known to dephosphorylate standard deoxynucleotide monophosphates (dNMPs) and can also act as a triphosphatase on dNTPs, as well as on some close nucleotide analogues^43,44^. After purification of the S-2L HD phosphohydrolase overexpressed in *E. coli*, we tested its activity by pre-incubating it with the reactional mixture for the aforementioned DNA polymerization assay, before adding PrimPol. We observed that the presence of the phosphohydrolase prevented polymerisation with dATP, but did not affect the polymerisation with dZTP (Fig. 2d).

**Fig. 4:**
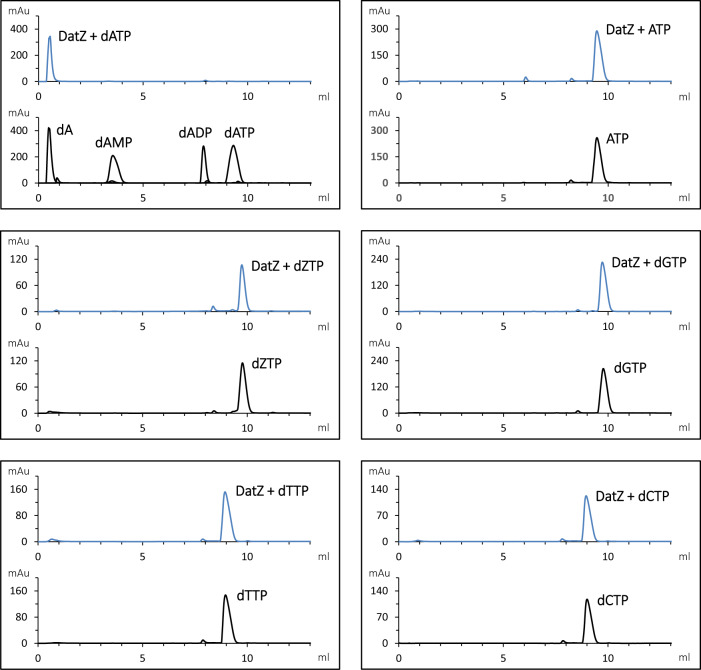
HPLC analysis of S-2L DatZ dephosphorylation products. Nucleotide standards are in black, products eluted after incubation of the corresponding triphosphates with DatZ are in blue. Each sample was eluted separately, using an amount of 40 nmol. The enzyme is active exclusively with dATP and removes from it all phosphates: it is therefore a triphosphohydrolase specific of dATP, or dATPase.

We interpreted this behaviour as the result of a specific dATP triphosphohydrolase activity, therefore suggesting to call the enzyme DatZ. We confirmed this hypothesis by incubating DatZ with different nucleotide triphosphates and analysing the reaction products by HPLC analysis (Fig. 4). dATP was rapidly degraded into dA; however, under the same conditions there was no dephosphorylation of ATP, dZTP, nor of all other standard dNTPs (dGTP, dTTP or dCTP). We also found no dephosphorylase activity on dADP or dAMP substrates (Supplementary Fig. 3a). Marginal tri-dephosphorylation products of dZTP start to appear only after a prolonged incubation (75x longer than for dATP) or in excess of DatZ concentration. Contrary to OxsA phosphohydrolase^44^, we did not observe a sequential dephosphorylation, but a one-step reaction directly from dNTPs to dNs, never detecting any intermediate phosphorylation states in the course of the reaction.

Our finding that S-2L DatZ is a specific dATP triphosphohydrolase offers a simple explanation of how the phage avoids incorporating adenine in its genome.

### DatZ structure at 0.86 Å resolution: general description

Using X-ray crystallography, we determined three structures of S-2L DatZ with its substrate, the reaction product and the metal cofactors, the second one at sub-angstrom resolution. They constitute the first structures of a viral HD phosphohydrolase, and the third HD phosphohydrolase to be described in atomic details, after *E. coli* YfbR^45^ and *B. megaterium* OxsA^44^.

**Fig. 5:**
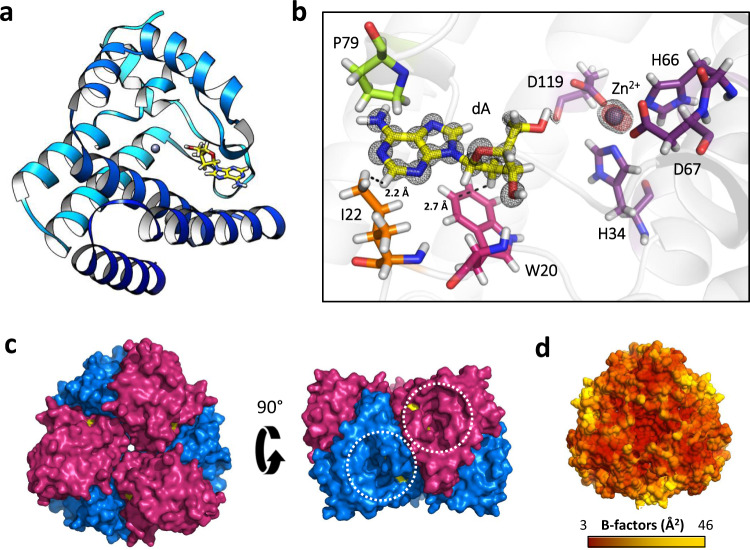
Three-dimensional structure of S-2L DatZ. **a** Ribbon representation of a DatZ monomer in a light blue-dark blue gradient, with bound dA in stick (yellow). The Zn^2+^ ion is shown as a grey sphere. **b** A close-up on the catalytic pocket of DatZ with the experimental 2F_o_-F_c_ electron density contoured at 2.5 sigmas around bound ligands: dA and Zn^2+^ (black mesh). Additionally, the anomalous density at Zn^2+^ absorption edge (red mesh) is contoured at 10 sigmas. Residue I22 (orange) provides direct specificity towards the adenine nucleobase, creating a steric hindrance for chemical groups in position 2 of the purine ring. Other residues highlighted in the text are Zn^2+^-coordinating ones (purple), W20 (magenta) and P79 (lime). **c** Structure of the full DatZ hexamer, top and side views, in surface representation. Blue and purple protomers form a compact, particularly stable disc in an alternating, zigzagging pattern. Two of the six symmetrical cavities leading to buried dA molecules (yellow) are visible in the side view and highlighted by the white dotted circles. **d** Surface representation of DatZ hexamer coloured by the experimental B-factors (dark red-yellow gradient, hydrogen atoms omitted), with the scale bar below. The highest temperature factors map to the flexible loop above dA.

First, we present a 0.86 Å resolution structure of S-2L DatZ bound to dA, the product of dephosphorylation of dATP in solution (PDB ID: 6ZPA; Supplementary Table 1). The electron density allowed to build the whole protein as well as 218 water molecules around the DatZ chain (175 aa), which is roughly the number expected for this resolution limit^46^. Although several hydrogen atoms are discernible at such a resolution, the usual limit for their experimental allocation is 0.8 Å^47^; they were therefore refined using a riding model. Each monomer of DatZ takes a globular form composed predominantly of α- and 3_10_-helices (70% and 4% respectively), with no β-strands (Fig. 5a). The base moiety of dA snugly fits in the catalytic pocket below a relatively flexible element (as indicated by higher B-factors), with the P79 residue on its tip (Fig. 5b). A catalytic divalent ion is found in the vicinity of dA’s free 5′-OH group, even though no divalent ion was added in buffers during purification or crystallisation. In the catalytic site, the side chain of residue I22 is ideally positioned to sterically exclude the amino group in position 2 of the purine ring of G or Z and provides an immediate explanation for the observed specificity of the enzyme. In addition, W20 side chain constitutes a steric hindrance for the 2′ hydroxyl group of any ribose-based nucleotide.

Concerning the oligomeric state of DatZ, we found that in crystallo it arranges in a compact toroidal hexamer with a D_3_ symmetry, where neighbouring subunits are flipped (Fig. 5c). Such a shape emerges from two partially hydrophobic, self-interacting protein sides (A:A and B:B), with a large surface of interaction – 1358.6 Å^2^ and 959.0 Å^2^. We confirmed the hexameric stoichiometry of DatZ in vitro with complementary techniques, i.e., DLS and analytical ultracentrifugation, leading to 5.9 (±0.1) protomers per oligomer assuming a perfectly globular shape. The whole hexamer is particularly rigid, as judged from the overall very low B-factors (Fig. 5d), which is consistent with the ultrahigh diffraction limit for DatZ crystals.

### A two-metal-ion mechanism of DatZ

In the literature, there is some ambiguity as to which divalent cation plays a catalytic role in HD phosphohydrolases. The structure of OsxA suggested the presence of one fixed Co^2+^ ion coordinated by the protein and one transient Mg^2+^ interacting with the triphosphate^44^. The YfbR enzyme was shown to be active with Co^2+^ and less with Mn^2+^, Cu^2+^ and Zn^2+^ (ref. ^43^), while OxsA is roughly equally active with Co^2+^, Co^2+^/Mg^2+^ and Mn^2+^, but not Zn^2+^ (ref. ^44^).

In S-2L DatZ, the first detected metal ion occupying the site “A” in the 0.86 Å resolution structure is coordinated by residues H34, H66, D67 and D119; two water molecules, also present in the Co^2+^-bound structure (see below), complete a typical octahedral coordination shell and fit well into the electron density map. Both the position and coordination of ion A^2+^ are identical to what is observed in other known HD phosphohydrolases, that take their name from the conserved HD diad. An excitation x-ray energy scan showed a major contribution of Zn; additionally, an anomalous double-difference signal at 40 sigmas at the Zn edge unambiguously point to the presence of a Zn^2+^ ion in this site. Its coordination geometry is less common than the usual tetrahedral one, but not atypical^48^. The fact that no additional divalent ions were added during protein purification indicates a high affinity of DatZ for Zn^2+^. Zn^2+^ is present in *E. coli* grown on LB medium^49^ at a level comparable to the one found in vivo in cyanobacteria^50^.

We then solved a second structure of DatZ co-crystallised with dATP and 10 mM CoCl_2_ (PDB ID: 6ZPB; Supplementary Table 1 and Supplementary Fig. 4a) and noticed the presence of a second, previously undescribed metal ion binding site, that we call “B”. This site is not the one observed in OxsA structure, although it lies in the vicinity of the first site (5.2 Å apart) as well. Both Co^2+^ ions are coordinated octahedrally: in site A, the binding geometry is the same as described above for Zn^2+^, while in site B the coordination is mediated by residues E70, D75, the O5′ of dA and three water molecules. The presence of the two Co^2+^ ions was confirmed by a strong signal in the corresponding Fourier double-difference anomalous map at 46 and 33 sigmas in sites A and B, respectively.

Finally, we solved a third structure of DatZ, this time with bound dATP (PDB ID: 6ZPC; Supplementary Table 1 and Supplementary Fig. 4b) but no divalent ion(s), obtained by adding EDTA to the enzyme before crystallising it with the triphosphate. In this structure, we could observe the residues K81 and K116 neutralising the negative charge of β- and γ-phosphates. We still find a Zn^2+^ ion in the A-site as shown by its anomalous signal, although not fully occupied and only penta-coordinated. We assume that this change in coordination, intermediate between tetrahedral and octahedral and also commonly observed for Zn^2+^^48^, is the result of the presence of a triphosphate.

**Fig. 6:**
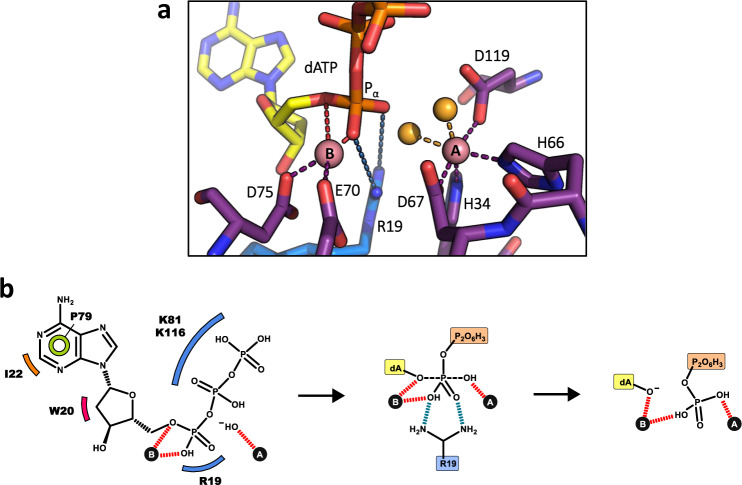
Catalytic centre of S-2L DatZ with the substrate and cofactors and the mechanism of tri-dephosphorylation. **a** Model of the reaction centre made by superposition of two of the structures solved in this work. The first structure defines dATP (in yellow) and residue R19 interacting with the α-phosphate (blue); hydrogen atoms were omitted for clarity. The second structure provides catalytic ions A and B (magenta spheres), bound water molecules that are likely to take part in the reaction (gold) and the metal coordinating residues (purple). Interacting atoms, ions and groups of interests are shown by dashed lines of corresponding colour. The distance between the two Co^2+^ ions is 5.2 Å. **b** Schematic diagram of DatZ reaction under two-metal-ion mechanism with the initial substrates, intermediate and products. Bonds being made and broken are shown in dashed lines; ionic interactions are in hashed red (with ionic cofactors) and blue (with protein). Interactions of the substrate with base-stabilising P79 (lime), sugar-specificity-conferring W20 (magenta), 2-amino-specificity-conferring I22 (orange), and triphosphate-neutralising K81 and K116 (blue) residues are additionally highlighted. In this diagram, a hydroxide ion (OH^−^) is proposed for the nucleophile.

Superposition of the new structures with both cofactors (divalent ions) and the substrate allows to propose a complete catalytic mechanism of DatZ (Fig. 6). Similarly to alkaline phosphatase and 3′−5′ exonuclease^51^, DatZ uses a typical two-metal-ion mechanism to dephosphorylate dATP. While the ion B^2+^ stabilises the leaving O5’ atom and one oxygen of the α-phosphate (P_α_), ion A^2+^ positions a hydroxide (OH^−^) in an attacking position opposite to O5’. Then, by interacting with OH^−^, the α-phosphate passes through a penta-coordinate intermediate, forming an unstable oxyanion stabilised by the R19 residue. Finally, the bond O5′-P_α_ is broken and a new one, P_α_-OH, is created.

We checked by HPLC that DatZ is active in a buffer containing Mg^2+^ as the sole added divalent metal ion and we observed that the enzyme stays active in crystallo with no additional divalent ions at all. Two additional crystal structures showed that Zn^2+^ in site A is replaced by Co^2+^ in excess of the latter (20 mM CoCl_2_), but is retained in elevated Mg^2+^ concentrations (50 mM MgSO_4_), as determined through anomalous signal analysis (see Methods).

### The active site of DatZ: conservation and mutagenesis

A number of phages that contain a close homologue of *purZ* gene in their genome also contain a homologue of *datZ*. Looking for the conservation of residues crucial for both a dATPase activity and absence of dZTPase activity, as identified by the present structural studies, we built a multialignment of these closely related DatZ sequences (Supplementary Fig. 5). We found that all residues stabilising both catalytic metal ions are strictly conserved, as well as R19, K81 and K116 interacting with α-, β- and γ-phosphates. Residues W20, I22 and P79, interacting with the base, are conserved or involve conservative substitutions. Additionally, residues Q29, A32 and G74 are strictly conserved among close DatZ homologues, highlighting their possible importance for protein structure (tertiary or quaternary) and/or its dynamics.

With the intention of engineering a dNTPase with a selectivity shifted towards dZTP, we cloned, expressed and tested DatZ I22A mutant, designed to make room for the additional amino group of Z in the binding pocket (Supplementary Fig. 6). We observed a significant relaxation of the purine specificity (Fig. 2d and Supplementary Fig. 3b). The mutant’s dATPase activity is clearly reduced and still does not show any intermediate product. The additional space created for the 2-amino group of dZTP has the desired effect of raising the dZTPase activity to the point of becoming detectable, albeit still very low. The dGTPase activity remains undetectable, indicating that the selectivity towards an amino group in position 6 of the purine ring is maintained.

## Discussion

The immediate neighbours of PrimPol in the S-2L genome are also replication-related proteins (exonuclease VIII, SF2 helicase and VRR nuclease; Supplementary Table 2), and all have a high level of sequence identity with Mediterranean uvMED phages’ corresponding proteins. In contrast, these viruses contain neither *purZ* nor *datZ* genes – they share with S-2L only their replicative machinery, and not the additional apparatus that enables the A-to-Z switch. Interestingly, S-2L PrimPol is also related to cyanobacterial enzymes: notably, sequence motifs in the AEP polymerase core correspond perfectly to these of All3500-like family^19^, with almost all of the high-scoring matches coming from cyanobacteria genus. Such a finding supports the idea that *pplA*, the gene of PrimPol, may have been exchanged between cyanophages and their hosts.

Due to the divergent nature of the AEP superfamily, its classification is far from trivial. The universal presence of its members, encompassing all three domains of life, viruses and plasmids, testifies about its ancient origin^19^. Advanced sequence-based computational methods divided the superfamily into four clades: AEP proper, NCLDV-herpesvirus primase, PrimPol, and BT4734-like^17^. In another approach using sequence clustering, AEPs were distributed into multiple groups, with the newly defined PrimPol-PV1 supergroup^19^. S-2L PrimPol belongs to the *Anabaena* (*Nostocaceae*) All3500-like (sub)family within the PrimPol clade or the PrimPol-PV1 supergroup, depending on the classification.

A search with PrimPol in the Dali server^52^ identified all structures of AEP available in the PDB. However, due to excessive divergence of the superfamily, the structure-based multialignment approach, applied below for DatZ, was not reliable. Instead, we adopted the geometry-based analysis proposed by Dali. Both the dendrogram and the non-hierarchical clustering method (Supplementary Fig. 7) distinguish two major, well-defined groups: archaeo-eukaryotic replicative PriS primases and bacterial NHEJ primases (LigC/D), belonging to the *AEP proper clade* defined previously^17^. The remaining set contains PrimPols with more distant homology. The strongest link between S-2L PrimPol and any other member of the AEP superfamily is with the plasmidic RepB′ (3H20), highlighting the connection between All3500-like and RepB′ clusters within the PrimPol-PV1 supergroup^19^. Additionally, the previously undescribed subfamily of AEP conserved in the order *Campylobacteriales* and represented by HP0184 from *H. pylori* (2ATZ) is systematically placed together with them, hinting that they may share a common ancestor.

In general, in spite of the modest set size of 15 unique AEP structures, PrimPols are clearly much more widespread and diverse than the PriS and NHEJ primases, which have more specific roles. Our preliminary analysis suggests that the ancestor of S-2L PrimPol was acquired from its cyanobacterial host.

Concerning DatZ, we performed a multialignment of all available HD phosphohydrolase structures with PROMALS3D (Supplementary Fig. 8), thus avoiding purely sequence-based errors. There is a strict conservation of all residues binding metal ion A across all representatives, along with metal B-binding E70 residue and R19 that stabilises the reaction intermediate. There are two singular cases where the D75 B-site binding residue can change to E or H, but chemically both are capable of metal ion coordination. Prominently, the human HD phosphohydrolase HDDC2 (HD domain-containing protein 2) shows a metal coordination identical to the one seen in S-2L DatZ; it is the only other homologue structure with two ions (Mg^2+^) present in both sites A and B (PDB ID 4DMB). Although it was hypothesised that during the nucleophilic attack a glutamic acid corresponding to DatZ E70 would act as a proton donor through a water bridge^45^, here we provide evidence that it participates in metal B binding instead. Interestingly, its alanine mutant was described as having lost its phosphohydrolase activity. Lastly, the residue E93 is almost completely structurally conserved, with the only exception of OxsA, and its position along the sequence is shifted one α-helix turn in DatZ; it remained undetected by previous sequence alignments with close viral DatZ homologues probably due to an intrinsic low precision in this region without structural support. E93 places its side chain in the catalytic pocket, but too far away to interact directly with the phosphate γ or the divalent metal ion B^2+^ (6.5 and 7.8 Å, respectively). We suggest that this glutamic acid may instead facilitate the free phosphates’ trafficking between the catalytic pocket and the solvent.

Using the multialignment data, we constructed a structurally informed phylogenetic tree of HD phosphohydrolases (Supplementary Fig. 9). Aside from following the typical distribution into the tree domains of life, it suggests that the ancestor of DatZ was acquired from a bacterial variant; the closest DatZ homologues found in BLAST represent the phyla of γ-proteobacteria and firmicutes (excluding the immediate viral clade), in conformity with this hypothesis.

Although diverse in sequence, the monomeric structures of the other known HD phosphohydrolases are very similar to DatZ (Supplementary Fig. 10a), with an average RMSD on C_α_ atoms of 2.75 Å. Despite the fact that only a dimer was described for related bacterial HD phosphohydrolases^44,45^, we discovered that the same hexameric quaternary state could be found by generating their symmetry-related mates using the space-group symmetry operators (Supplementary Fig. 10b). In fact, a high multimeric state (>3) has been also reported in vitro for YfbR^43^, compatible with our hypothesis.

As all residues crucial for the reaction in DatZ are conserved or replaced by similar residues in other structures, we suggest that the two-metal-ion mechanism described above is universal for all HD phosphohydrolases, completing previous reports by the identification of metal ion site B and correcting the role of residue E70 counterparts (Supplementary Fig. 10c). Interestingly, OxsA replaced the positively charged K116 with E129 bearing negative charge; we propose that it is this exception that facilitates the accommodation of a third divalent metal ion observed in OxsA and absent in DatZ, which efficiently neutralises the charge of the triphosphate.

In conclusion, we note that the strategy adopted by the phage S-2L phage is most probably shared with related phages containing homologous *datZ* and *purZ* genes. It is very similar to the strategy adopted by the T2, T4 and T6 phages that contain a substantial amount of hydroxymethylcytosine, relying on a dCTP triphosphatase to also shift the pool of available dNTPs in their host cell^6^.

In the future, it will be interesting to see if *datZ* and *purZ* genes are sufficient for transferring 2-aminoadenine to the genomes of other organisms.

## Methods

### Identification of genes of interest

The genomic sequence of cyanophage S-2L was obtained from NCBI’s database (AX955019). Potential ORFs were identified using ORFFinder^53^ (>150 nt, genetic code 11). Targeted ORFs were assessed for possible homology with known proteins using BLAST. The genomic positions of genes involved in phage replication is provided in Supplementary Table 2; nucleotide sequences of native and codon-optimised genes *pplA* and *datZ* are specified in Supplementary Tables 3 and 4. Protein disorder of PrimPol was predicted with DISOPRED^27^.

### Protein expression and purification

Synthetic genes for expressed proteins were optimised for *E. coli* and synthesised using ThermoFisher’s GeneArt service. Genes were cloned into modified pRSF1-Duet expression vector with a TEV-cleavable N-terminal 14-histidine tag^54^ using New England Biolabs and Anza (Thermo Fisher Scientific) enzymes. Shorter versions of PrimPol (PP300 and PP190) were cloned by adding overhangs with codon STOP and corresponding cleavage site through standard PCR with designed oligonucleotides (Eurogentec); mutagenesis of DatZ was done using designed oligonucleotides and QuikChange II Site-Directed Mutagenesis Kit (Agilent). *E. coli* BL21-CodonPlus (DE3)-RIPL cells (Agilent) were separately transformed with engineered plasmids. Bacteria were cultivated at 37 °C in LB medium with appropriate antibiotic selection (kanamycin and chloramphenicol), and induced at OD = 0.6–1.0 with 0.5 mM IPTG. After incubation overnight at 20 °C, cells were harvested and homogenised in suspension buffer: 50 mM Tris-HCl pH 8, 400 mM NaCl, 5 mM imidazole. After sonication and centrifugation of bacterial debris, corresponding lysate supernatants were supplemented with Benzonase (Sigma-Aldrich) and protease inhibitors (Thermo Fisher Scientific), 1 μl and 1 tablet per 50 ml, respectively. Proteins of interest were isolated by purification of the lysate on Ni-NTA column (suspension buffer as washing buffer, 500 mM imidazole in elution buffer). They were further diluted to 150 mM NaCl and repurified on HiTrap Heparin (for PrimPol) or HiTrap Q (for DatZ) columns (1 M NaCl and no imidazole in elution buffer). Histidine tags were removed from the proteins by incubation with his-tagged TEV enzyme overnight. After removing TEV on Ni-NTA column, proteins were further purified on Superdex 200 10/300 column with 25 mM Tris-HCl pH 8, 150 mM NaCl (for PrimPol-N190 crystallisation a 16 mM concentration of NaCl was used). All purification columns were from Life Sciences. Protein purity was assessed on an SDS gel (BioRad). The enzymes were concentrated to 7–19 mg ml^−1^ with Amicon Ultra 10k and 30k MWCO centrifugal filters (Merck), flash frozen in liquid nitrogen and stored directly at −80 °C, with no glycerol added. Selenomethionine (SeMet) version of PP-N190 was prepared using the same expression strain and construct. Bacteria grew in medium with 6 g L^−1^ Na_2_HPO_4_, 3 g L^−1^ KH_2_PO_4_, 1 g L^−1^ NH_4_Cl, 0.5 g L^−1^ NaCl, 2 mM MgSO_4_, 100 μM CaCl_2_ and 0.4% glucose, supplemented with metal solution (5000x): 5 g L^−1^ FeCl_2_, 184 mg L^−1^ CaCl_2_, 64 mg L^−1^ H_3_BO_3_, 40 mg L^−1^ MnCl_2_, 18 mg L^−1^ CoCl_2_, 4 mg L^−1^ CuCl_2_, 340 mg L^−1^ ZnCl_2_, 605 mg L^−1^ Na_2_MoO_4_, 1.3 μl L^−1^ and 0.8% conc. HCl. At OD = 0.6, cultures were supplemented with 50 mg L^−1^ of selenomethionine, isoleucine, leucine and valine, and 100 mg L^−1^ of lysine, threonine and phenylalanine. All chemicals were from Sigma-Aldrich. After further incubation for 15 min at 37 °C, the culture was induced and processed as above.

### DNA polymerase assays

Radioactivity-based polymerase activity tests, if not stated otherwise for a particular condition, were executed in 200 mM Tris-HCl pH 8 and 50 mM MgCl_2_, with 50 nM of dT_10_GG overhang DNA template, 50 nM of α-32P 5′-labelled DNA primer complementary to template upstream sequence, 250 μM dNTP mix and 1 μM of PrimPol (20 min of incubation) at 37 °C. Fluorescence-based polymerase activity tests, if not stated otherwise, were executed in 20 mM Tris-HCl pH 7 and 5 mM MgCl_2_, with 3 μM of dT_12_ overhang DNA template, 1.5 μM of FAM 5′-labelled DNA primer, 500 μM dNTP mix, 0.5 µM of PrimPol constructs (10 min of incubation) and 1 µM of DatZ (8 min) at 37 °C. The Klenow polymerase used as a control was at 5 U in 50 μl (10 min incubation). Polymerase gene replication test was conducted similarly, with 3 μM of template and primer labelled radioactively on 5′ end (α-32P); PP-300 at 42.3 μM and Klenow polymerase at 4 U in 20 μl were incubated for 15 min. Oligonucleotide sequences are specified in Supplementary Table 5.

Before adding the protein, DNA was hybridised by heating up to 95 °C and gradually cooling to reaction temperature. Reactions were terminated by adding two volumes of a buffer containing 10 mM EDTA, 98% formamide, 0.1% xylene cyanol and 0.1% bromophenol blue, and stored in 4 °C. Products were preheated at 95 °C for 10 min, before being separated with polyacrylamide gel electrophoresis and visualised by FAM fluorescence or radioactivity on Typhoon FLA 9000 imager. All oligonucleotides were ordered from Eurogentec, chemicals from Sigma-Aldrich, Klenow polymerase from Takara Bio, standard dNTPs from Fermentas (Thermo Fisher Scientific) and dZTP from TriLink BioTechnologies.

### Nucleotide HPLC analysis

In all, 1 µM of DatZ or its mutant was incubated at 37 °C for 10 min with 500 μM of the respective dNTP, in a buffer containing 20 mM Tris pH 7 and 5 mM MgCl_2_. Reaction products were separated from the protein using 10 000 MWCO Vivaspin−500 centrifugal concentrators and stored in −20 °C. Products and standards were assayed separately, using ~40 nmol of each for anion-exchange HPLC on DNA-PAC100 (4 × 50 mm) column (Thermo Fisher Scientific). After equilibration with 150 μl of a suspension buffer (25 mM Tris-HCl pH 8, 0.5% acetonitrile), nucleotides were injected on the column and eluted with 3 min of isocratic flow of the suspension buffer followed by a linear gradient of 0–200 mM NH_4_Cl over 10 min (1 ml min^−1^). Eluted nucleotides were detected by absorbance at 260 nm, measured in arbitrary units [mAu]. High-purity nucleotides and chemicals were bought from Sigma-Aldrich, and HPLC-quality acetonitrile was from Serva.

### Crystallography and structural analysis

All crystallisation conditions were screened using the sitting drop technique on an automated crystallography platform^55^ and were reproduced manually using the hanging drop method with ratios of protein to well solution ranging from 1:2 to 2:1. PrimPol-N190 was screened at 14.5 mg ml^−1^ in 4 °C. Elongated rods grew over 2 days in 100 mM CaCl_2_, 20% w/v PEG 8k (40%) and 5% v/v isopropanol (100%) buffered with 100 mM MES pH 6. DatZ was screened at 12–17 mg ml^−1^ with a molar excess of 1.2 of dATP at 18 °C. Big, symmetric crystals grew rapidly over 1–2 days in 1.5 M Li_2_SO_4_ buffered with 100 mM HEPES pH 7.5. All crystals were soaked in a solution containing 70% crystallisation buffer and 30% glycerol and frozen in liquid nitrogen. Crystallographic data was collected at the SOLEIL synchrotron in France (beamlines PROXIMA-1 and PROXIMA-2), processed by XDS^56^ with the XDSME^57^ pipeline and refined in Phenix^58^. Nucleotide constraints for structure refinement and dZ modelling were obtained using Grade Web Server^59^. The structure of PrimPol-N190 was solved by SAD technique using SeMet derivative of the protein and datasets collected at the selenium edge (0.9807 Å) using the SHELX C/D/E programmes^60^. The structure of DatZ was solved by the sulphur-SAD (S-SAD) technique at 1.7712 Å wavelength. The anomalous double-difference Fourier map for Zn was calculated from data collected at 9.67 and 9.66 keV (Zn peak and pre-edge). DatZ ultrahigh resolution structure was obtained by merging 3 individual datasets taken on the same crystal. Structures of DatZ with bound Co^2+^ and dATP were obtained by growing crystals with 10 mM CoCl_2_ and 10 mM EDTA, respectively (the latter at pH 7). Replacement of the Zn^2+^ ion by Co^2+^ was confirmed with anomalous double-difference maps with data collected at 7.73 and 7.28 keV (Co peak and pre-edge wavelengths). We found two major peaks at 46.4 sigma and 32.7 sigma at sites A and B, respectively. Retention of the Zn^2+^ ion in presence of Mg^2+^ was confirmed with a persisting strong anomalous signal at 7.1 keV, 12.7 keV and 16 keV.

### Molecular dynamics simulations of PP-N190

Force field parameters of dCTP were obtained using CGenFF^61^. The parameter penalty and the charge penalty were zero, indicating that the parameters can be used safely without any modification. CHARMM36 parameter set was used for the rest of the system^62^. Topologies of the structures were prepared with psfgen module of VMD^63^. After the topology construction, the structures were solvated in a triclinic box with a distance of at least 11 Å to the box edges and TIP3P solvent model. The systems were neutralised with Na^+^ and Cl^−^ ions, and the ion concentration was set to 0.15 M. Then, a 50,000 step conjugate gradient minimisation procedure was carried out. The minimised systems were heated up to 300 K with 0.001 K steps. An NPT equilibration procedure followed the heating. The equilibration time was 2 ns and the time step was 2 fs. The equilibration temperature (300 K) was controlled with Langevin thermostat and the pressure (1 atm) was controlled with Langevin barostat. The production run was 212 ns long, with the remaining parameters of production runs identical to the equilibration stage parameters. All of the molecular dynamics simulations were performed with NAMD version 2.13^64^.

### Sequence and structure alignments, phylogeny

Close relatives of *pplA* and *datZ* were identified by BLAST searches, and aligned with Clustal Omega^65^ (PrimPol) or the default MUSCLE algorithm in MEGA X software^66^ (DatZ); sequence logos were made with WebLogo^67^. Structures homologous to PrimPol and DatZ available in PDB were identified using Dali server^52^; Dali was further used for pairwise RMSD determination and geometry analysis. The tendencies observed for AEP superfamily clustering were maintained whether the analysis involved whole structures or only AEP cores, and whether the dataset was complete or not. The sequences of DatZ and other structures from HD phosphohydrolase family were aligned in PROMALS3D^68^ using structural data supplemented by full protein sequences, excluding not-superimposable N- and C-termini. Multialignment images were prepared with ESPript 3^69^. Maximum-likelihood phylogenetic tree of HD phosphohydrolases based on their structural multialignment was prepared in MEGA X with default parameters, taking 100 bootstrap replications. All protein structures were visualised with Chimera^70^ and Pymol^71^.

### Statistics and reproducibility

All non-crystallographic experiments and molecular dynamics simulations were done in triplicates (*n* = 3). For x-ray crystallography, several consistent datasets were collected from multiple crystals; the best-resolution datasets were chosen for the final refinements.

### Reporting summary

Further information on experimental design is available in the Nature Research Reporting Summary linked to this paper.

## Supplementary information

Supplementary Information

Reporting summary

## Data Availability

The crystallographic data for proteins PP-N190 and DatZ bound to various ligands are deposited in the Protein Data Bank (PDB) under the accession codes 6ZP9 [10.2210/pdb6ZP9/pdb], 6ZPA [10.2210/pdb6ZPA/pdb], 6ZPB [10.2210/pdb6ZPB/pdb] and 6ZPC [10.2210/pdb6ZPC/pdb]. Raw images and data used to generate the figures and plots are provided in the Source Data file. Other data are available from the corresponding author upon request. Source data are provided with this paper.

## Source data

Source Data
