# Supplementary material for: How cyanophage S-2L rejects adenine and incorporates 2-aminoadenine to saturate hydrogen bonding in its DNA
Source: Nat Commun. 2021 Apr 23;12:2420. doi: 10.1038/s41467-021-22626-x (PMC8065100; doi:10.1038/s41467-021-22626-x)
Supplement: Supplementary file 1 — Supplementary Information [file 41467_2021_22626_MOESM1_ESM.pdf]

# How cyanophage S-2L rejects adenine and incorporates 2-aminoadenine to saturate hydrogen bonding in its DNA

Dariusz Czernecki, Pierre Legrand,
Mustafa Tekpinar, Sandrine Rosario,
Pierre-Alexandre Kaminski and Marc Delarue

# Supplementary Information

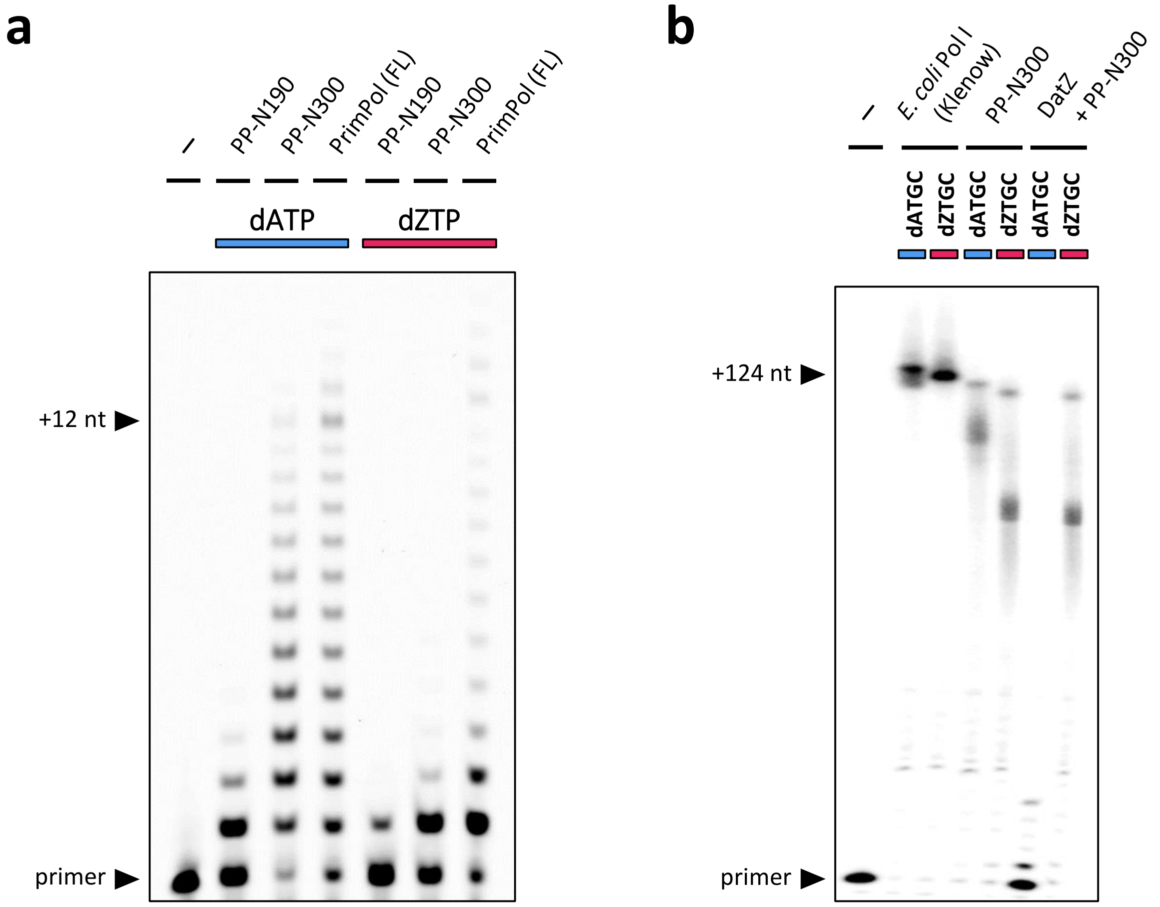


**Supplementary Figure 1. Additional polymerase activity tests on S-2L PrimPol constructs, using nucleotide dATP/dATGC mix (blue) or dZTP/dZTCG mix (magenta). a** Polymerisation assay of the three PrimPol constructs, with a negative control without any polymerase in the first lane. The incubation was conducted for 20 min, using the $dT_{12}$ overhang template. **b** Polymerisation assay for the first 124 nt of PrimPol's native gene. Results are shown for a negative control without any polymerase (lane 1), a positive control with *E. coli* Pol I (Klenow fragment) (lanes 2-3), PP-N300 polymerase without (lanes 4-5), or with pre-incubation of the reactional mixture with DatZ (lanes 6-7). The polymerisation step was allowed to proceed for 15 min with 42.3 µM of PP-N300.

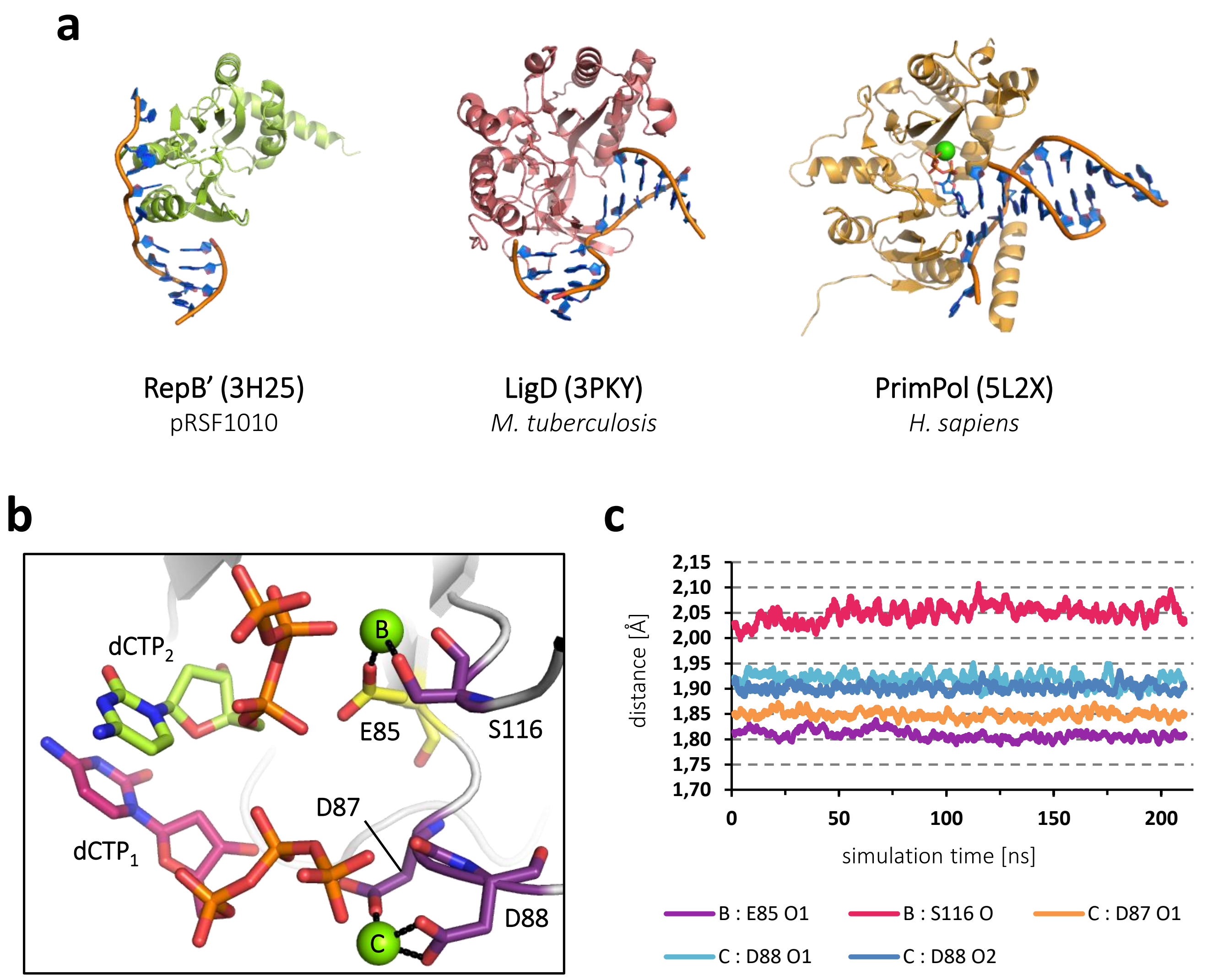


**Supplementary Figure 2. AEP and its ligands. a** Three known AEP structures with bound dsDNA, viewed from the same perspective. Below are: the protein name, PDB code of the structure and the organism or plasmid of origin. The DNA molecule seems to bend in an L-shape at the catalytic site. **b** A model of PP-N190 with two $Mg^{2+}$ ions (green) bound in B and C sites and two nucleotides in the initiation (magenta) and elongation (lime) sites, obtained after energy minimization step. Residues interacting with $Mg^{2+}$ ions in a way previously undescribed are in purple, the ones interacting in a typical way – in pale yellow. The ionic bonds are visualised by the black lines. **c** Distances between the residues and bound ions shown in (b), with the colour code given below the graph. They were measured in the course of 212 ns of the simulation and averaged in the 2 ns frame. The novel ion in site C interacts with the γ-phosphate of the nucleotide in the initiation site; the binding is stable and similar across all simulations.

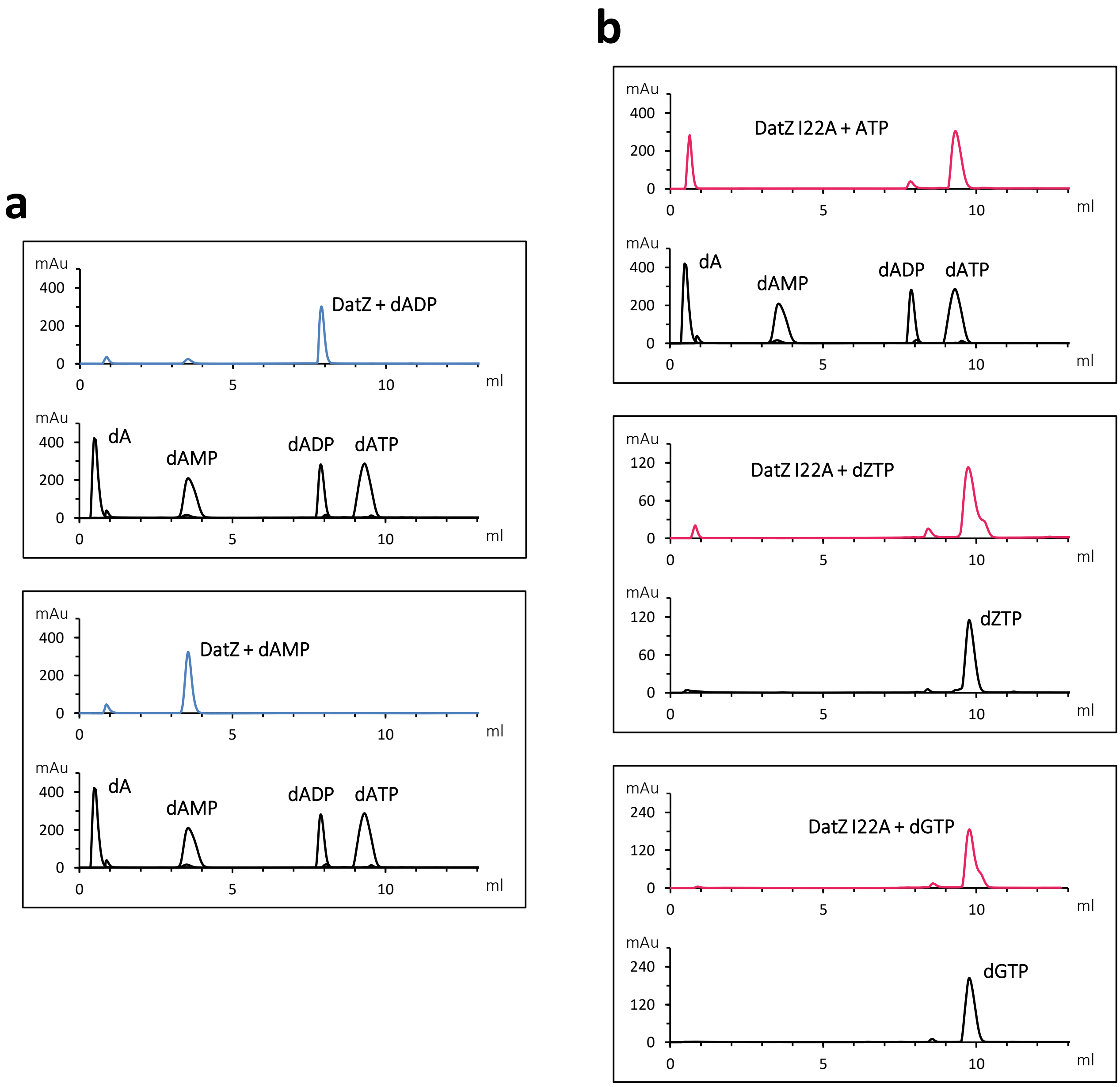


**Supplementary Figure 3. Further tests on S-2L DatZ catalytic activity and its I22A mutant.** The panels are constructed as in Figure 4. **a** HPLC analysis of nucleotides obtained after incubation of DatZ with dADP and dAMP, showing no discernible dephosphorylation products. **b** Same analysis for dATP, dZTP and dGTP incubated with DatZ I22A. Compared to the wild-type enzyme, the mutant shows reduced dATPase activity and improved, although still marginal, dZTPase activity. dATP to dA tri-dephosphorylation occurs in a single step.

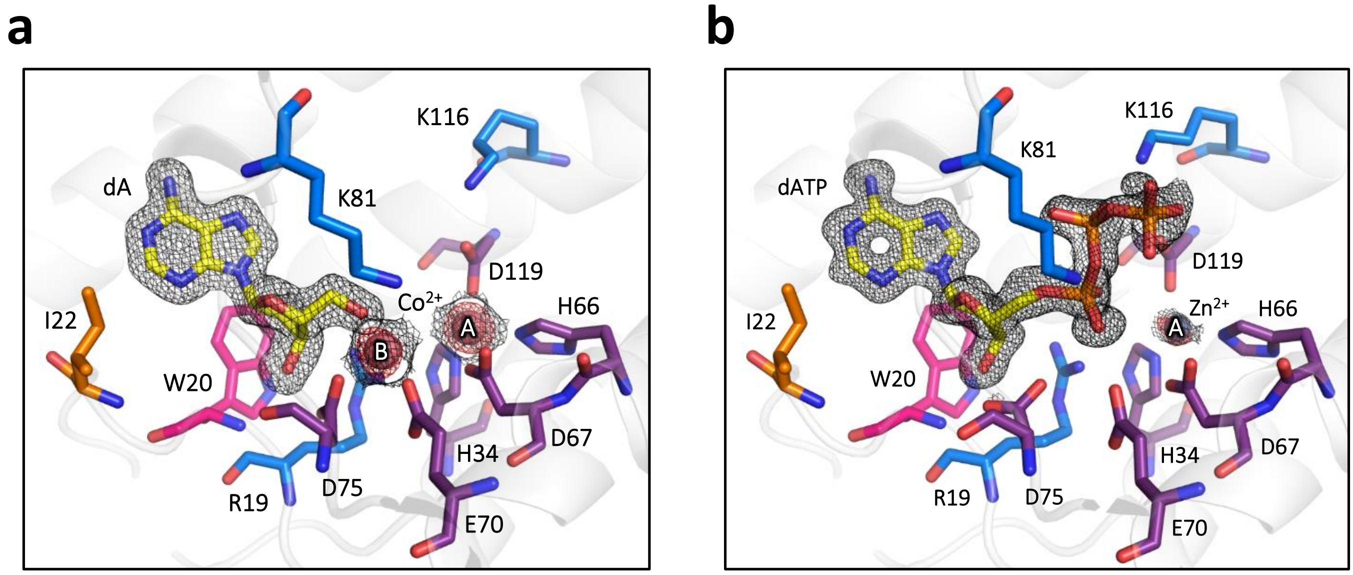


**Supplementary Figure 4. Catalytic centre of S-2L DatZ dATPase with bound substrate, product and cofactors.** Colour code as in Figure 5b and Figure 6a; residues K81 and K116, balancing the charge of the triphosphate, are also displayed. Water molecules and hydrogen atoms are omitted for clarity. **a** Structure of DatZ with dA and $Co^{2+}$. The 2Fo-Fc electron density map is contoured at 1 sigma around dA and $Co^{2+}$ ions in the binding sites named A and B (black mesh). The anomalous signal at the wavelength of data collection is contoured at 10 sigmas (red mesh). **b** Structure of DatZ with dATP and partially occupied $Zn^{2+}$, using the same representation. The 2Fo-Fc electron density map is contoured at 1 sigma around dATP and $Zn^{2+}$ ion in the binding site A (black mesh). Residual amounts of pentacoordinated $Zn^{2+}$ can be identified by the anomalous signal at Zn edge, contoured here at 5 sigmas (red mesh).

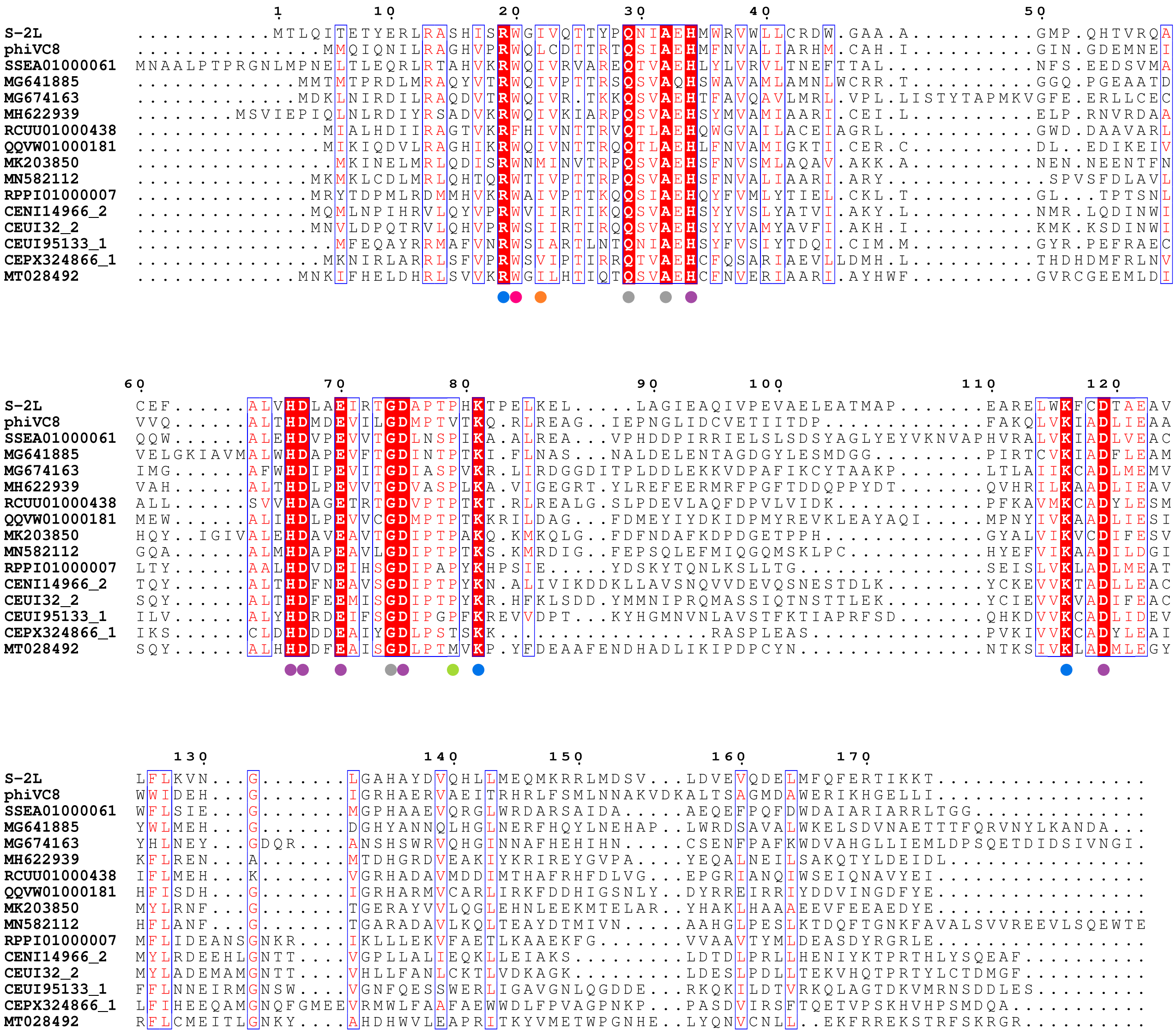

**Supplementary Figure 5. Sequence multialignment of close DatZ homologues co-occurring with purZ gene in related phages.** Numbering above the alignment refers to S-2L DatZ. Dots below the alignment mark positions of residues crucial for DatZ: residues coordinating metal ions A and B (purple); residues stabilising the triphosphate (blue); W20 discriminating ribonucleotides (magenta); I22 providing steric hindrance for Z and G nucleobases (orange); P79 stabilizing the purine ring (lime). The remaining strictly conserved residues with hypothetical structural importance are marked by a grey dot.

Because of large inconsistencies in naming, phages other than S-2L and ϕVC8 are described by their reference number (left). They correspond to (in order): *Sinobacteraceae* bacterium, phage PMBT28, phage SH-Ab 15497, *Siphoviridae* sp. ctbf_3, Compost metagenome, *Bacteroidetes* bacterium, phage ZP6, *Podoviridae* sp. ctpVR23, *Chloroflexi* bacterium, four unnamed sequences from Tara database and phage vB_OspP_OH. The supposedly bacterial sequences are short and match other viral sequences on the full length of the genome.

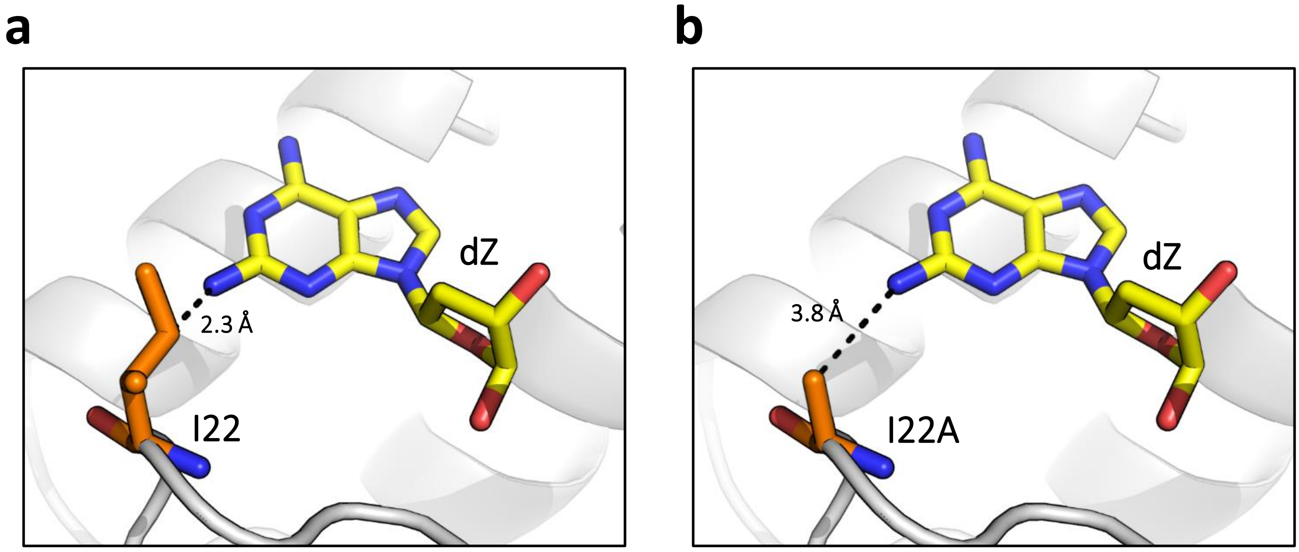


**Supplementary Figure 6. A model of dZ in the catalytic pocket of DatZ and its mutant I22A. a** The distance between dZ nitrogen atom of the amino group in position 2 and the closest atom of I22 ($C_\gamma$) is shown by a dashed line. It is too short to allow for correct dZ binding. **b** The distance between the same nitrogen atom and the closest atom of the side chain in mutant I22A ($C_\beta$) is longer by 1.5 Å (dashed line).

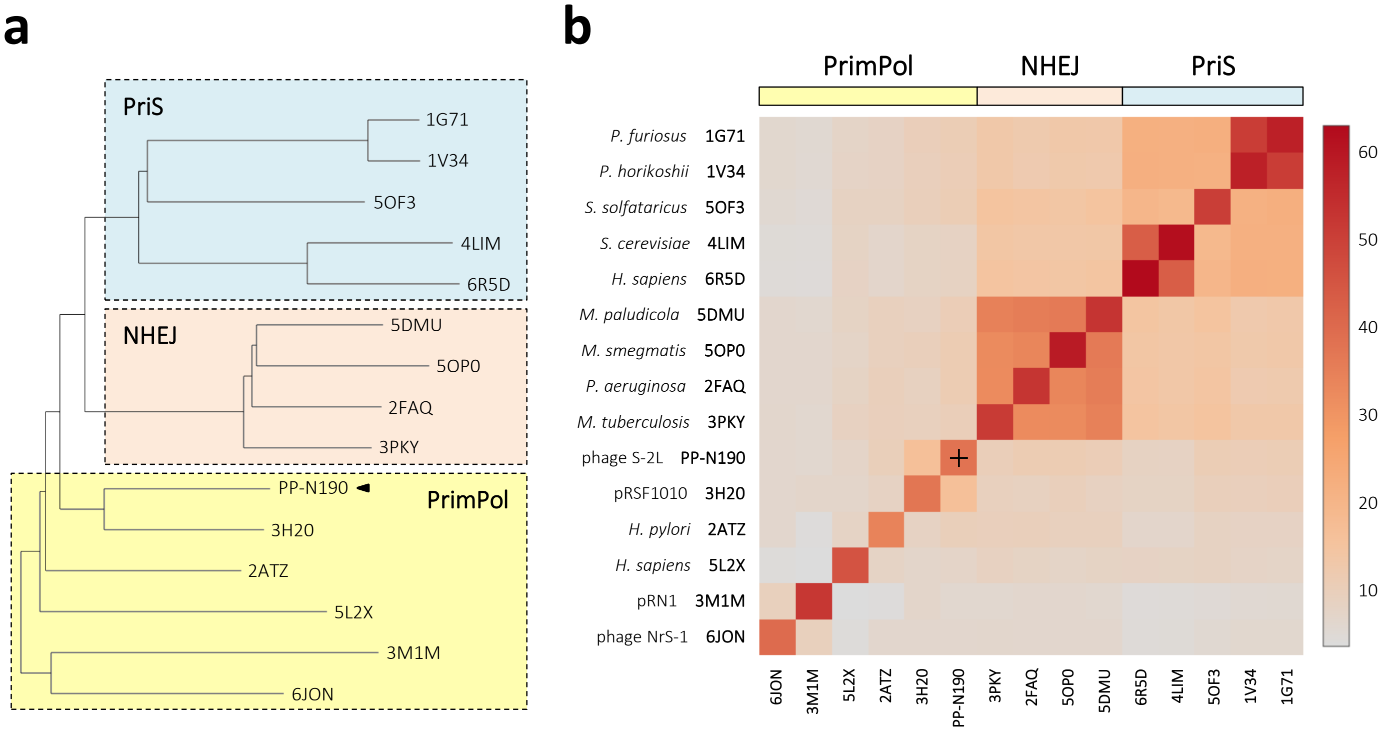


**Supplementary Figure 7. Structural classification of available AEP enzymes with Dali. a** Dendrogram of AEP superfamily, derived by hierarchical clustering of the similarity matrix data. PDB codes are atop of the branches; PP-N190 is marked by a black triangle. Archaeo-eukaryotic PriS (light blue) and bacterial NHEJ primases (light orange) are monophyletic, and group together in so-called AEP proper clade. Primase-Polymerases (PrimPols, light yellow) are more divergent and spread-out across all three domains of life, viruses and plasmids. The AEP domain of S-2L PrimPol shares a recent ancestor with plasmidic RepB'. **b** The similarity matrix data. PDB codes are indicated to the left and below, organism names further to the left, protein families above and similarity scale to the right. PP-N190 is highlighted with a black cross. Each square represents with its colour how close structurally a pair of AEP proteins is, varying from grey (no similarity) to dark red (high closeness).

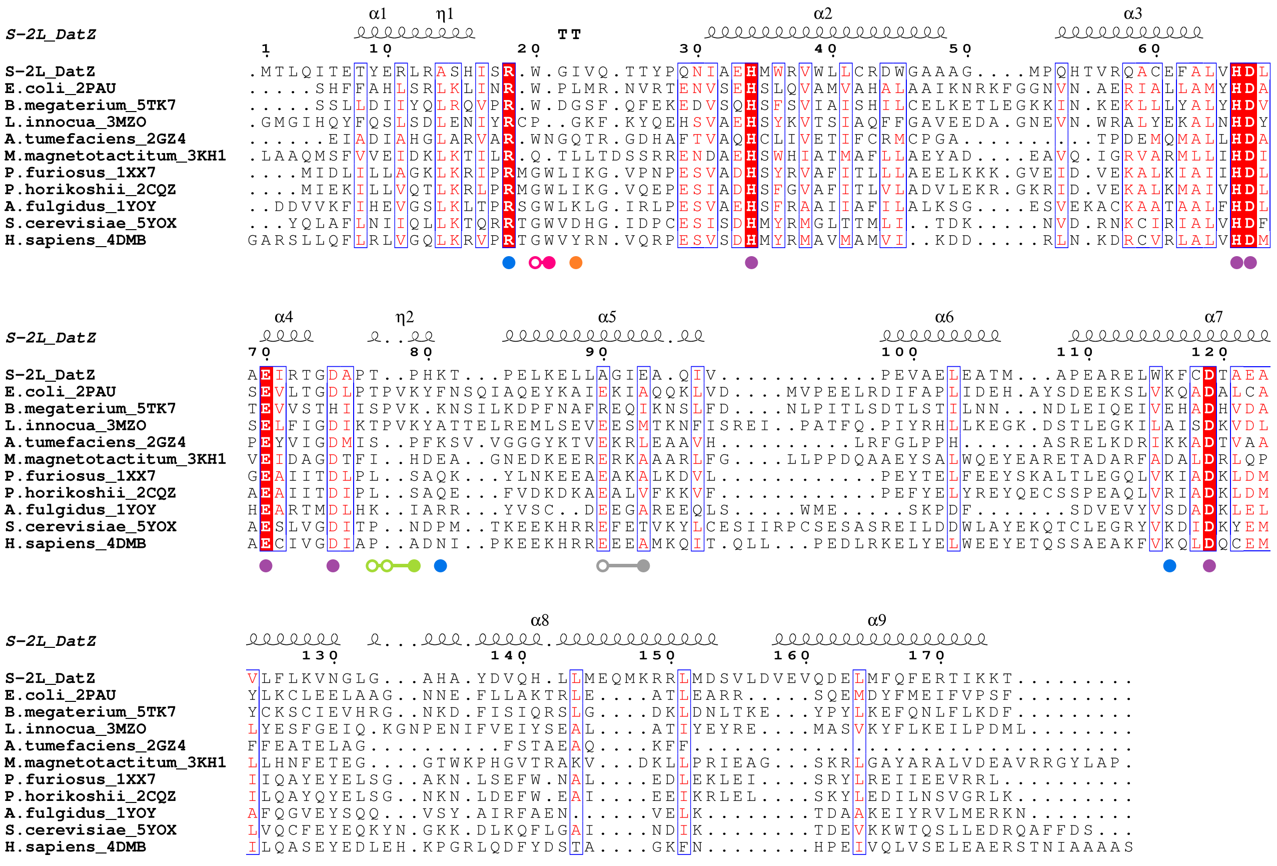


**Supplementary Figure 8. Structural multialignment of S-2L DatZ and all other HD phosphohydrolases whose crystal structure is available in the PDB.** Organism names and PDB codes are indicated on the left. The meaning of the coloured dots below the alignment is the same as in Supplementary Figure 5; the additional conserved residue E93 is marked by a grey dot. Empty circles highlight highly conserved residues with slightly shifted backbone positions with respect to S-2L (connected full circles), but with superimposed similar functional groups. Occasional unstructured and unbuilt regions in the middle were aligned using sequence information alone while non-superposable N- and C-termini were ignored; in particular, an extended, but structured N-terminus of *L. innocua* and the last α-helix of *A. tumefaciens* that undergoes a considerable positional shift, were omitted.

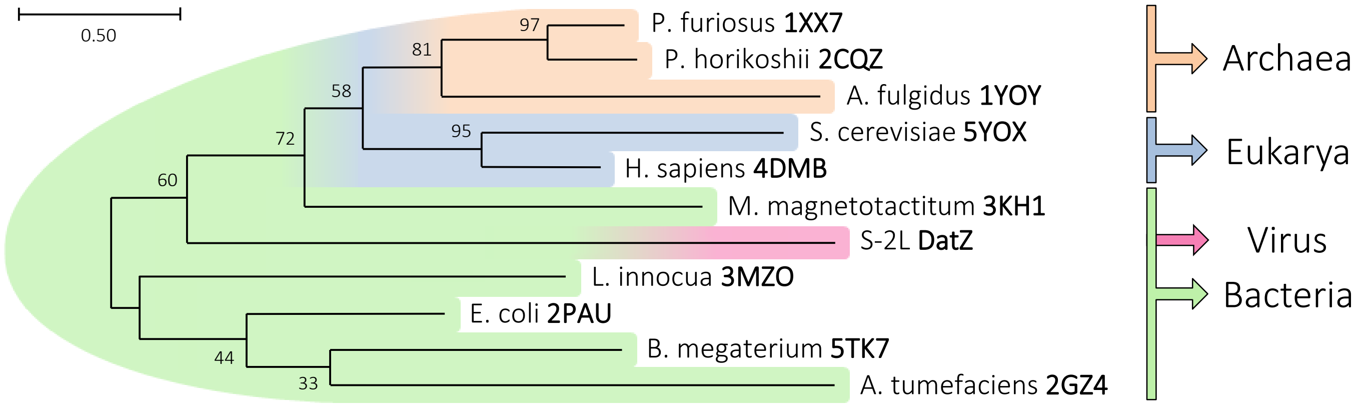


**Supplementary Figure 9. Non-rooted maximum-likelihood phylogenetic tree of HD phosphohydrolases for all available molecular structures.** The tree was calculated using the alignment from the Supplementary Figure 8. Organism names and PDB codes are to the right of the corresponding branches. Enzymes divide into three groups: archaeal, eukaryotic and bacterial/S-2L, suggesting an acquisition of the datZ gene by S-2L's ancestor from a bacterium. Numbers on the nodes are bootstrap percentage values; the reference distance corresponds to an average 0.5 substitution per site. The topology of the bootstrap consensus tree is identical, supporting the result.

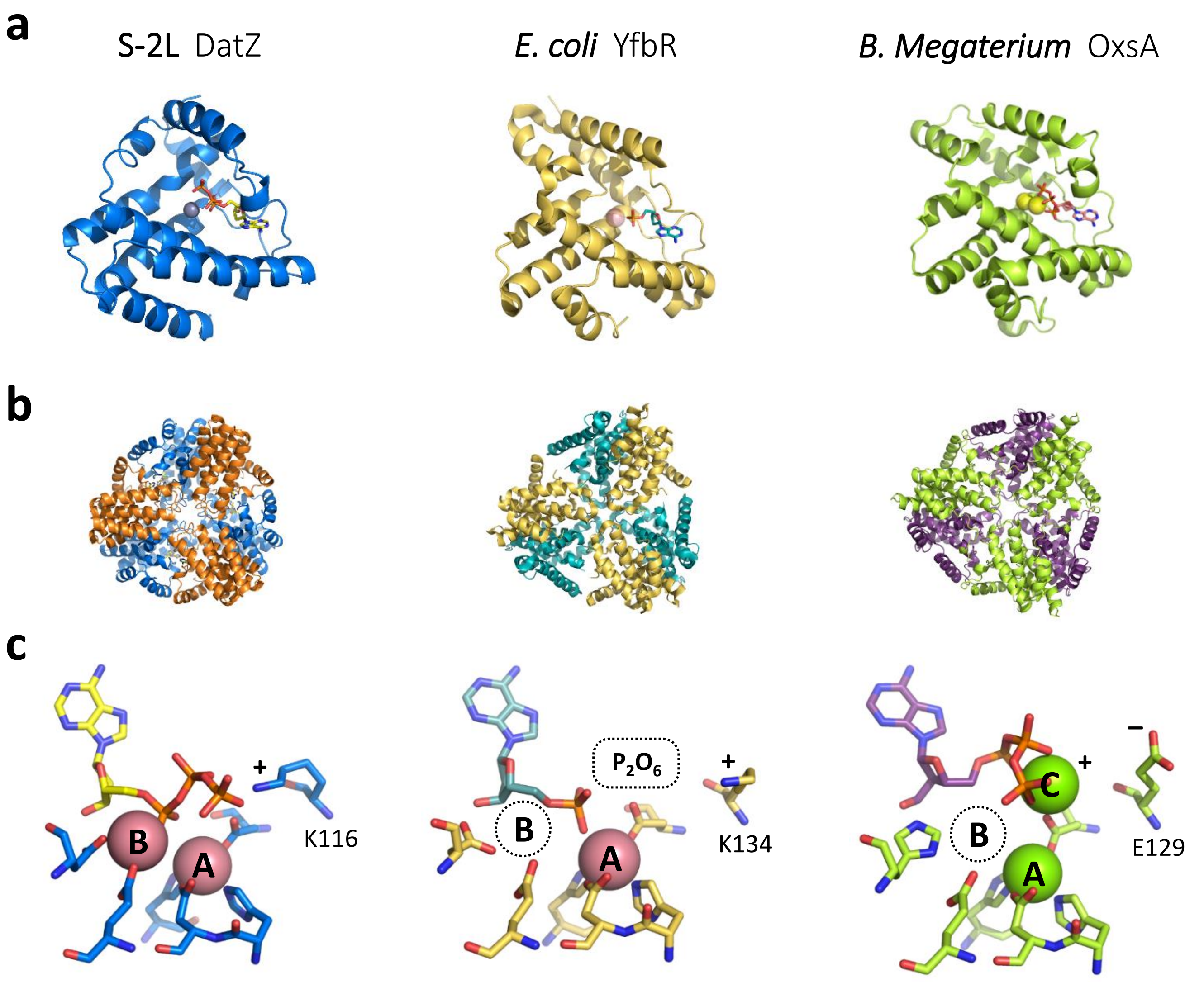


**Supplementary Figure 10. Common structural features across the HD phosphohydrolase family.** Comparison of S-2L DatZ with *E. coli* YfbR and *B. Megaterium* OxsA. **a** Structures 6ZPC, 2PAU (chain A) and 5TK7. The overall protein fold is highly conserved, with RMSD of 2.0 Å and 2.3 Å, respectively, as well as the position of substrates and fixed catalytic divalent ions. **b** Hexameric quaternary organisation of DatZ, YfbR and OxsA, extrapolated by using crystallographic symmetry operators to generate the hexamer. **c** Comparison of the reaction centre between S-2L DatZ and *E. coli* or *B. megaterium* HD phosphohydrolases. DatZ is represented as in Figure 6a; Yfbr E72A mutant structure with bound dAMP is taken from PDB 2PAU, with A72 residue swapped with natural E72 from PDB 2PAQ; OxsA is from PDB 5TK7. Features missing in YfbR and OxsA models can be inferred from DatZ structures (shown by dotted contours), suggesting that the metal ion site B is universal and has similar coordination across the whole protein family. Site C, observed for OxsA, would be a result of a switch from mostly conserved positively charged residue corresponding to DatZ's K116 to a negatively charged one, justifying the need for a third divalent cation.

| **Protein structure** | **PrimPol-N190** | **DatZ + dA, $Zn^{2+}$** | **DatZ + dA, $Co^{2+}$** | **DatZ + dATP, $Zn^{2+}$** |
|---|---|---|---|---|
| PDB ID | 6ZP9 | 6ZPA | 6ZPB | 6ZPC |
| *Cell parameters* | | | | |
| Space group | P $2_1$ | R 3 2 | R 3 2 | R 3 2 |
| *a*, *b*, *c* (Å) | 59.2, 47.7, 66.2 | 141.5, 141.5, 53.6 | 141.8, 141.8, 53.7 | 141.7, 141.7, 53.7 |
| *α*, *β*, *γ* (°) | 90.0, 97.1, 90.0 | 90.0, 90.0, 120.0 | 90.0, 90.0, 120.0 | 90.0, 90.0, 120.0 |
| Solvent content (%) | 41.5 | 51.5 | 51.9 | 51.7 |
| *Data statistics* | | | | |
| Resolution (Å) | 47.69 - 1.50<br>(1.54 - 1.50) | 49.11 - 0.86<br>(0.87 - 0.86) | 40.44 - 1.72<br>(1.77 - 1.72) | 40.89 - 1.27<br>(1.30 - 1.27) |
| Wavelength (Å) | 0.980097 | 0.729309 | 1.033202 | 0.980100 |
| Rmerge (%) | 10.2 (140.0) | 10.5 (474.9) | 8.3 (30.7) | 6.6 (128.7) |
| Completeness (%) | 96.8 (94.6) | 100.0 (100.0) | 98.4 (88.0) | 99.8 (97.7) |
| Multiplicity | 7.1 (7.0) | 61.2 (51.8) | 14.2 (5.7) | 19.5 (17.6) |
| $I/\sigma(I)$ | 11.8 (1.4) | 27.6 (1.7) | 25.0 (5.1) | 21.5 (2.0) |
| $CC_{1/2}$ | 0.999 (0.555) | 1.000 (0.689) | 0.999 (0.939) | 1.000 (0.686) |
| *Refinement* | | | | |
| Resolution (Å) | 41.30 - 1.50 | 40.34 - 0.86 | 40.44 - 1.72 | 35.41 – 1.27 |
| Unique reflections | 56,610 | 172,367 | 21,608 | 54,249 |
| $R_{work}/R_{free}$ (%) | 16.57/17.27 | 12.92/13.48 | 14.60/17.02 | 12.58/14.61 |
| *No. of non-hydrogen atoms* | | | | |
| Protein | 2953 | 1455 | 1451 | 1435 |
| Ligand | 0 | 18 | 18 | 30 |
| Ions | 3 | 2 | 3 | 3 |
| Water | 552 | 218 | 260 | 231 |
| Hydrogen atoms | No | Yes | No | Yes |
| *Protein geometry* | | | | |
| RMSD - bond lengths (Å) | 0.007 | 0.006 | 0.011 | 0.008 |
| RMSD – bond angles (°) | 0.85 | 0.94 | 1.29 | 0.95 |

| **Protein structure** | **PrimPol-N190** | **DatZ + dA, $Zn^{2+}$** | **DatZ + dA, $Co^{2+}$** | **DatZ + dATP, $Zn^{2+}$** |
|---|---|---|---|---|
| PDB ID | 6ZP9 | 6ZPA | 6ZPB | 6ZPC |
| *Protein geometry (cont.)* | | | | |
| RMSD - bond lengths (Å) | 0.007 | 0.006 | 0.011 | 0.008 |
| RMSD – bond angles (°) | 0.85 | 0.94 | 1.29 | 0.95 |
| Ramachandran favored/outliers (%) | 100.00/0.00 | 99.42/0.00 | 98.83/0.00 | 99.42/0.00 |
| Rotamers favored/poor (%) | 96.13/0.32 | 96.84/0.00 | 98.72/0.00 | 98.06/0.00 |
| Clashscore | 2.91 | 2.36 | 1.70 | 1.02 |
| *B-factors ($Å^2$)* | | | | |
| Type | Anisotropic | Anisotropic | Isotropic | Anisotropic |
| Protein | 19.86 | 11.72 | 15.96 | 19.15 |
| Ligand | - | 9.80 | 12.04 | 36.68 |
| Ions | 22.66 | 9.66 | 15.00 | 20.24 |
| Water | 34.78 | 25.21 | 26.55 | 34.25 |

Supplementary Table 1. Diffraction data collection and model refinement statistics. Numbers in parenthesis refer to the highest-resolution shell.

| Protein name | Function | Start | End |
|---|---|---|---|
| PrimPol | DNA polymerase | 20924 | 23134 |
| DatZ | dATPase | 14035 | 13511 |
| Exonuclease VII | exonuclease | 18069 | 17113 |
| Helicase SF2 | helicase | 18971 | 20233 |
| VRR nuclease | resolvase | 20246 | 20752 |

Supplementary Table 2. Position of replication-related protein genes on S-2L reference genome (AX955019).

| Gene | Nucleotide sequence |
| --- | --- |
| *pplA* | ATGTCAACCCCCGCACCAGCCTTCGACCGGGACCAGATCCTCCTCCACCTGTCGCTCCTCCGGAAGGACATCGCCACGACCCGGTACCG<br>GGCGATCTGGCCCAGGCGAGAGGACAAGGTAAAAGCCTGGACGACGCCCCTGACCGGGGCCACGGTCCAGGACGCCGTCACCCAGGGAT<br>TCAACAGCTACATCGTCGTAGGCGACGGCGGCGACTCCGACGCCGAGATCACCAGTGTCAACGCCATCTTCGGCGAGTGGGACGACGGC<br>GACCTGGCCTGGCAGGTCGGCGCCTGGGAGGCCTGCGGCCTGCCGCGGCCGAGCTTCCAGCTGCGCACCGGGGGCAAGTCGATCCACCA<br>CTACTGGGTGTTCCACAGCCCTGTGGACGTCCCGGCCTGGACCGAGCTCCAGGCCCGCCTGATCGCCCTGGCCGGCTTCGACACGACGA<br>ACCGGAACCCCTCCCGGGTGATGCGCCTGGCCGGCTGCCCCCACCAGCGCACCGGGGAGGTGGCCCAGATCTTCAACGCGACCGGGGAG<br>CTCTACGACCCCGGGCAGATGCTGCAGGTCCTGCCCCCGGTGCCGATCGACCCGCCGGCTGCCGCCCCGGTGGCCCCGGGAGGTGCCCC<br>CAGTTCGATGGACGACATCCGGGCCGCCCTGGCCCAGATCCCACCCCGTCCCGGGGCAGGGAGCGGCACCTACGCCGAGTACCGCAACA<br>TCCTCTGGGGCCTGGTTAAGGCCGTCGAGGAGGCCGGCGGCACCCGGGACCAGGCCGTGGCCATGATGCAGGCGCACAGCCCCGAGGGC<br>TGGGATTGCGCCCAGGTGGCCCGCTCCGGGGGCAAGAAGATCAGCACCGGGACGTTCTGGTGGCATGCGATGTCCTACGGCTGGGCACC<br>GCCGAAGAAGGCCCCGGAGCCGCCGCCCCAGGCCCGCCAGGTGCCGGCCGTGGCCGCCGTGCTCCAGGCCGCAGAGGCCGCCCCTGGAA<br>CCGGCACCGAGCACGGCCCCTGGGCGCCGCTGCCCCCGGGCTGGCAGGGCACGAACAAGGAGGGCCTGCCCAGGGCCTCGCAGATCACC<br>ACCTACGAACTGGCCCTGCTGATGCAGGTCTCCCTGCGGGGGGTGCTCTGGCACAACGAGATGTCAGGCGAAGTCATGCACGGCAAGAC<br>GGCCCTCTCGCCGATCGAGCTCCAGATCGCCTACAGCCGGCTCGAGGGCCTCGGCTACAAGGTCACCAAGGAGAACGCCAAGACCGCCA<br>TCCTGCAGGCGTCGATCGCCGACCTGCGGCACCCCGTCCGGGAGTACCTCAACACCTGCACGACGCCCCTGCCCGACGAGGTCTGGGCC<br>GACATCGCCAACGCCCTGCTGGGCCCCGGGCACAGCGCGTTCGACTCCAGCGCCATCCGCAAGTGGCTGATCTTCGCCGTGGCCCGGGT<br>CTTCCAGCCCGGCTGCCCCTTCGGCTTCATGCTGGTGCTGGCTGGCGCCCAGCAGATGCACAAGACCCGGTTCTTTAACACCCTGGCCT<br>CAGACGAGTGGTTCCTGGGCGGATTCCAGCGGGGCCGCTCTGACACCGACGACCTGATTGCCCTGCACCGGTCCTGGATCACCGAGTGG<br>GGGGAGCTCGACGGCGGCCTCTCCAAGCACGACAGCGCCGAGCTCAAGGCGATGATCGACCGGAAGGTGGACGTGCTCCGGAGGCCCTA<br>CGCCGCCACGCACGAAAGCTGCCCCCGGAGCTTCGTCCTCTGCGGGACGACGAACCGCCGGGATGGGCTCTTCACCGACCCGACCGGCA<br>ACAGGCGGTACGTGGTCGTGCCCGTCAACCAGCGGATCGACAGCGAGCGCCTGGAGCAGATGCGAGACCAGATCTGGGCAACCGCCCTC<br>CGGGAGTACCGCAGCGGCAAGCTCTGGTACCTCGACGAGGAGGAGCTGGAGATCAACGCGAAACGCAACAAGGGCCTTGAGGTGGAGGA<br>CGCCTGGGTGGGGACGATCCAGATGCACCTGAATAGCTCGATCGACCTGGAGCGGCTGACCGACGGGCGCTACGGCATCAACATCGAGT<br>CAGTCTACCTCAAGATCGAGCCCGAGGTGGGACGCCGTGGCCCGGGCTTCGGAAAGCGGATCCGGGACACCATGCTGAGCCTGGGCTGG<br>GAGCCCGTGCGGCTGCGTCTCGCCAGCGACCCGAGCGGCAACCCGGTGAGGCGTTGGGCGCCCGTCCAGGGGGGGTAG |
| *datZ* | ATGACACTCCAGATCACCGAGACCTACGAGCGCCTGAGGGCGTCCCACATCAGCCGGTGGGGATCGTCCAGACGACCTACCCGCAGAA<br>CATCGCCGAACACATGTGGCGCGTTTGGCTCCTGTGCCGGGACTGGGGCGCTGCCGCCGGCATGCCCCAGCACACGGTCCGCCAGGCCT<br>GCGAGTTTGCCCTGGTCCACGACCTGGCCGAGATCCGGACGGGCGACGCCCCGACGCCCCACAAGACCCCGGAGCTCAAGGAGCTCCTG<br>GCCGGCATCGAGGCCCAGATCGTCCCGGAGGTGGCCGAGCTCGAGGCGACCATGGCCCCGGAGGCCAGAGAGCTTTGGAAGTTCTGCGA<br>CACCGCCGAGGCCGTCCTGTTCCTCAAGGTCAACGGCCTGGGCGCCCACGCCTACGACGTCCAGCACCTGCTGATGGAGCAGATGAAAC<br>GGCGCCTGATGGACTCGGTGTTGGATGTGGAGGTGCAGGACGAGCTCATGTTCCAGTTCGAGCGGACGATCAAGAAGACGTGA |

Supplementary Table 3. Nucleotide sequences of *pplA* and *datZ* native genes.

| Gene | Nucleotide sequence |
|---|---|
| *pplA* | ATGAGCACACCGGCACCGGCATTTGATCGTGATCAGATTCTGCTGCATCTGAGCCTGCTGCGTAAAGATATTGCAACCACACGTTATCG<br>TGCAATTTGGCCTCGTCGTGAAGATAAAGTTAAAGCATGGACCACACCGCTGACCGGTGCAACCGTTCAGGATGCAGTTACCCAGGGTT<br>TTAATAGCTATATCGTTGTTGGTGATGGTGGTGATAGTGATGCAGAAATTACCAGCGTTAATGCCATTTTTGGTGAATGGGATGATGGT<br>GATCTGGCATGGCAGGTTGGTGCATGGGAAGCATGTGGTCTGCCTCGTCCGAGCTTTCAGCTGCGTACCGGTGGTAAAAGCATTCATCA<br>TTATTGGGTTTTTCACAGTCCGGTTGATGTTCCGGCATGGACCGAACTGCAGGCACGTCTGATTGCACTGGCAGGTTTTGATACCACCA<br>ATCGTAATCCGAGCCGTGTTATGCGTCTGGCAGGCTGTCCGCATCAGCGCACCGGTGAAGTTGCACAGATTTTCAATGCAACCGGTGAA<br>CTGTATGATCCGGGTCAGATGCTGCAGGTTCTGCCTCCGGTTCCGATTGATCCGCCTGCAGCAGCTCCGGTTGCGCCTGGTGGTGCACC<br>GAGCAGCATGGATGATATTCGTGCAGCACTGGCACAGATTCCGCCTCGTCCTGGTGCAGGTAGCGGCACCTATGCAGAATATCGTAATA<br>TTCTGTGGGGTTTAGTTAAAGCCGTTGAAGAGGCAGGCGGTACACGTGATCAGGCAGTTGCAATGATGCAGGCACATAGTCCGGAAGGT<br>TGGGATTGTGCACAGGTTGCACGTAGTGGTGGCAAAAAAATCAGCACCGGTACATTTTGGTGGCATGCAATGAGCTATGGTTGGGCACC<br>GCCTAAAAAAGCACCGGAACCGCCTCCGCAGGCACGCCAGGTTCCAGCAGTTGCAGCAGTTCTGCAGGCAGCAGAAGCAGCCCCTGGTA<br>CAGGCACCGAACATGGTCCGTGGGCTCCGCTGCCTCCTGGTTGGCAGGGCACCAATAAAGAAGGTCTGCCACGCGCAAGCCAGATTACC<br>ACCTATGAACTGGCACTGCTGATGCAGGTTAGCCTGCGTGGTGTTCTGTGGCATAATGAAATGAGCGGTGAAGTAATGCATGGTAAAAC<br>CGCACTGAGCCCGATTGAACTGCAGATTGCATATAGCCGTCTGGAAGGTCTGGGTTATAAAGTGACCAAAGAAAATGCAAAAACCGCAA<br>TCCTGCAGGCAAGCATTGCCGATCTGCGTCATCCGGTTCGTGAATATCTGAATACCTGTACAACCCCTCTGCCGGATGAAGTTTGGGCA<br>GATATTGCCAATGCACTGTTAGGTCCGGGTCATAGCGCATTTGATAGCAGCGCAATTCGTAAATGGCTGATCTTTGCAGTTGCACGTGT<br>TTTTCAGCCTGGTTGTCCGTTTGGTTTTATGCTGGTGCTGGCAGGCGCACAGCAGATGCATAAAACACGCTTTTTTAACACCCTGGCAT<br>CCGATGAATGGTTTTTAGGTGGTTTTCAGCGTGGTCGTAGCGATACCGATGATCTGATTGCCCTGCATCGTAGCTGGATTACCGAATGG<br>GGTGAACTGGATGGTGGTCTGAGCAAACATGATAGCGCAGAACTGAAAGCAATGATTGATCGTAAAGTTGATGTGCTGCGTCGTCCGTA<br>TGCAGCAACCCATGAAAGCTGTCCGCGTAGCTTTGTTCTGTGTGGTACAACCAATCGTCGTGATGGTCTGTTTACCGATCCGACCGGTA<br>ATCGTCGTTATGTTGTTGTTCCGGTTAATCAGCGTATTGATAGCGAACGTCTGGAACAAATGCGCGATCAGATTTGGGCCACCGCACTG<br>CGCGAATATCGTTCAGGTAAACTGTGGTATCTGGATGAAGAGGAACTGGAAATTAATGCCAAACGCAATAAAGGTCTGGAAGTTGAAGA<br>TGCATGGGTTGGCACCATTCAGATGCACCTGAACAGCAGCATTGATCTGGAACGTCTGACCGATGGTCGTTATGGTATTAACATTGAAA<br>GCGTGTACCTGAAAATTGAACCGGAAGTTGGTCGTCGTGGTCCTGGTTTTGGTAAACGTATTCGTGATACCATGCTGAGCTTAGGTTGG<br>GAACCTGTTCGTCTGCGCCTGGCAAGCGATCCGAGTGGTAATCCGGTGCGTCGCTGGGCACCTGTTCAAGGTGGTTAA |
| *datZ* | ATGACACTGCAGATTACCGAAACCTATGAACGTCTGCGTGCAAGCCATATTAGCCGTTGGGGTATTGTTCAGACCACCTATCCGCAGAA<br>TATTGCAGAACATATGTGGCGTGTTTGGCTGCTGTGTCGTGATTGGGGTGCAGCAGCAGGTATGCCGCAGCATACAGTTCGTCAGGCAT<br>GTGAATTTGCACTGGTTCATGATCTGGCAGAAATTCGTACCGGTGATGCACCGACACCGCATAAAACACCGGAACTGAAAGAACTGCTG<br>GCAGGTATTGAAGCACAGATTGTTCCGGAAGTTGCAGAACTGGAAGCAACCATGGCACCGGAAGCACGTGAACTGTGGAAATTTTGTGA<br>TACCGCAGAAGCAGTTCTGTTCCTGAAAGTTAATGGTCTGGGTGCACATGCATATGATGTTCAGCATCTGCTGATGGAACAAATGAAAC<br>GTCGTCTGATGGATAGCGTTCTGGATGTTGAAGTTCAGGATGAACTGATGTTTCAGTTTGAACGCACCATCAAAAAGACCTAA |

Supplementary Table 4. Nucleotide sequences of *pplA* and *datZ* codon-optimised genes.

| Oligonucleotide name | Sequence (5’-3’) |
|---|---|
| $T_{10}GG$ overhang template | GGTTTTTTTTTTAACAAGGCTAATGCG |
| $T_{12}$ overhang template | TTTTTTTTTTTTAACAAGGCTAATGCG |
| PrimPol first 124 nt native gene template | CTTTTACCTTGTCCTCTCGCCTGGGCCAGATCGCCCGGTACCGGGTCGTGGCGATGTCCTTCCGGAGGAG CGACAGGTGGAGGAGGATCTGGTCCCGGTCGAAGGCTGGTGCGGGGGTTGACATAACAAGGCTAATGCG |
| Universal primer | CGCATTAGCCTTGTT |

Supplementary Table 5. Oligonucleotides used for polymerase assays.
